# Characteristics, aetiology and implications for management of multiple primary renal tumours: a systematic review

**DOI:** 10.1038/s41431-024-01628-5

**Published:** 2024-05-27

**Authors:** Huairen Zhang, Avgi Andreou, Rupesh Bhatt, James Whitworth, Bryndis Yngvadottir, Eamonn R. Maher

**Affiliations:** 1 Department of Medical Genetics, School of Clinical Medicine, University of Cambridge, Cambridge, CB2 0QQ UK; 2 Department of Urology, Queen Elizabeth Hospital, Birmingham, B15 UK; 3 Aston Medical School, College of Health and Life Sciences, Aston University, Birmingham, B4 7ET UK

**Keywords:** Risk factors, Cancer genetics

## Abstract

In a subset of patients with renal tumours, multiple primary lesions may occur. Predisposition to multiple primary renal tumours (MPRT) is a well-recognised feature of some inherited renal cancer syndromes. The diagnosis of MPRT should therefore provoke a thorough assessment for clinical and genetic evidence of disorders associated with predisposition to renal tumourigenesis. To better define the clinical and genetic characteristics of MPRT, a systematic literature review was performed for publications up to 3 April 2024. A total of 7689 patients from 467 articles were identified with MPRT. Compared to all patients with renal cell carcinoma (RCC), patients with MPRT were more likely to be male (71.8% versus 63%) and have an earlier age at diagnosis (<46 years, 32.4% versus 19%). In 61.1% of cases MPRT were synchronous. The proportion of cases with similar histology and the proportion of cases with multiple papillary renal cell carcinoma (RCC) (16.1%) were higher than expected. In total, 14.9% of patients with MPRT had a family history of cancer or were diagnosed with a hereditary RCC associated syndrome with von Hippel-Lindau (VHL) disease being the most common one (69.7%), followed by Birt-Hogg-Dubé (BHD) syndrome (14.2%). Individuals with a known or likely genetic cause were, on average, younger (43.9 years versus 57.1 years). In rare cases intrarenal metastatic RCC can phenocopy MPRT. We review potential genetic causes of MPRT and their implications for management, suggest an approach to genetic testing for individuals presenting with MPRT and considerations in cases in which routine germline genetic testing does not provide a diagnosis.

## Introduction

Kidney cancer accounts for >400,000 cancer diagnoses globally per year and has been increasing in frequency over the past few decades [1]. In part this increase reflects the occurrence of incidental diagnoses during abdominal scanning for other indications such as musculoskeletal or gastrointestinal complaints [2]. Surgical resection with curative intent is performed for localised renal cell carcinoma (RCC) but around a third of patients present with advanced disease and up to 40% of those treated surgically will go on to develop distant metastases and/or recurrence [3].

RCC is a heterogeneous disorder which may be subclassified by histopathological and/or molecular findings [4]. The most frequent histopathological subtype is clear cell (conventional; CRCC) RCC (75%) followed by papillary RCC (PRCC) (15%), chromophobe RCC (ChRCC) (5%) and other rarer histological and molecular subtypes [4]. Oncocytomas are benign renal tumours. PRCC can be further subdivided into Type 1 and Type 2 subtypes [4]. Some histological subtypes are associated with characteristic molecular findings in the tumour (e.g. chromosome 3 loss and inactivation of the *VHL* tumour suppressor gene (TSG) in CRCC, copy number gains of chromosome 7 and activating *MET* protooncogene alterations in Type 1 hereditary papillary renal carcinoma (HPRC)) whereas other molecular alterations (e.g. mutations in *FLCN, SETD2*, *TP53* genes) can be a feature of both CRCC and non-CRCC subtypes [4, 5]. The increasing application of molecular genetic profiling has led to the recognition of rare subtypes that are defined by a specific pathogenetic mechanism e.g. fumarate hydratase (FH)-deficient RCC, succinate dehydrogenase (SDH)-deficient RCC (0.05–0.2%), *TFE3-*rearranged RCC, *TFEB*-altered RCC, *ELOC*-mutated RCC and *ALK*-rearranged RCC [6].

RCC usually presents as a single unilateral lesion but in 3–10% of cases multiple primary renal tumours (MPRT) occurs (defined by the occurrence of two or more tumours in one or both kidneys of either benign or malignant nature that have arisen independently) [7–9]. MPRT may be synchronous or metachronous (the second tumour presents more than 6 months after the first) [10–12] and the renal tumours may have the same or different histopathologies [13]. However, the definition of MPRT excludes cases in which there was a single primary renal cancer and intrarenal metastatic secondary tumours [14]. An association between MPRT and inherited renal cancer syndromes such as von Hippel-Lindau (VHL) disease has long been recognised, though in the many cases of MPRT no underlying genetic cause is apparent (for example in a clinical audit of diagnostic testing for a five-gene panel (*VHL, FLCN, MET, FH* and *SDHB*) in 39 patients with MPRT without a family history or syndromic features, no pathogenic germline variants were identified (unpublished observations; Andreou A). Recently, novel genetic causes and mechanisms of MPRT have been described and here we review these advances and undertake a systematic literature review of MPRT.

## Methods

The literature search was conducted in PubMed according to PRISMA guidelines. The search used combinations of the key words (“renal cell carcinoma” or “oncocytoma”) AND (“metachronous” OR “synchronous” OR “multifocal” OR “bilateral”) and included literature published up to 3rd April 2024. Only accessible English literatures of individuals with clear indication of MPRT were included. Animal studies, reviews and comments were excluded. Other renal tumours i.e., angiomyolipoma, Wilms tumour, papillary adenoma, renal myxofibrosarcoma, and mixed epithelial and stromal tumours were excluded for MPRT. Individuals with only one renal tumour, recurrent renal tumour, metastatic renal tumour, renal tumour with kidney failure, and those receiving haemodialysis, renal transplant, and heart transplant, and those exposed to carcinogens were also excluded. Individuals with other cancers that are not associated with hereditary RCC-associated syndrome were excluded. For duplicated cohorts only one instance was kept (Supplementary Fig. 1).

For the publications that met the inclusion criteria, data were inserted into a spreadsheet. This included the publication index in PubMed of the literature, the number of individuals with MPRT, their age (or mean age), gender, family history of cancer, clinical diagnosis of hereditary RCC-associated syndrome, synchronicity and histology of their MPRT, and the result of their genetic tests. The distribution of age at diagnosis of the first RCC in individuals with MPRT was displayed in a histogram. Individuals with MPRT were grouped based on their known or likely genetic causes, and their ages were compared using a *t*-test, and qualitative comparisons were assessed using a chi-squared test.

## Results

### Characteristics of MPRT: systematic literature review and data analysis

A total of 467 articles describing individual cases or cohorts of patients with apparent MPRT were identified and these were divided into two groups: firstly, those reporting aggregated data (“Group A”) and secondly, those with individual patient level data (“Group B”). In the former group the number of participants with MPRT in each individual study ranged from 2 to 1063 (median = 21) whilst in the latter group the number of cases per study ranged from 1 to 22 individuals (median = 1). Overall, there were 6958 participants from 141 reports in group A and 731 participants from 326 reports in group B (total 7689 individuals) (Supplementary Tables 1 and 2). It was noted that in some studies there was evidence of an ascertainment bias as in some publications unselected patients with MPRT were included, in others there was a focus on a specific subgroup of patients with MPRT (e.g. those with VHL disease). Supplementary Tables 1 and 2 include information on how patients were ascertained and data for synchronicity, RCC histology, family history, genetic status etc (when available).

**Fig. 1:**
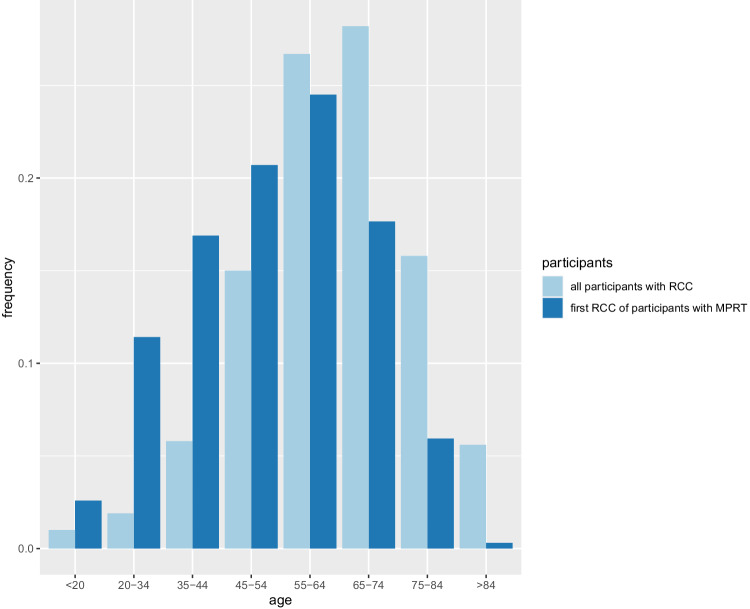
Age at diagnosis distribution for renal cell carcinoma. Age at diagnosis of renal cell carcinoma (RCC) in individuals with multiple primary renal tumours (MPRT) in the systematic review cohort (Group B) versus the age distribution for an unselected cohort of patients with RCC [16].

Analysis of all individuals with MPRT in both Group A and B showed that patients with MPRT were more likely to be male (71.8%, 3195/4452 with information available) versus the 63% reported for all RCC patients in the UK [15]. More patients with MPRT had synchronous (61.1%) than metachronous (38.9%) tumours. For those cases in which the age at diagnosis was available (Group B), there was a higher frequency of early age at diagnosis (≤46 years) of first RCC in patients with MPRT than that reported from a series of unselected patients with RCC (32.4% and 19% respectively) (see Fig. 1) [16]. In all patients with MPRT, 14.9% (430/2879)) had a family history of cancer or were diagnosed with a hereditary RCC-associated syndrome; however, some meta-studies only recruited patients with hereditary RCC-associated syndrome whereas few excluded them (see Supplementary Tables 1 and 2).

On average individuals with MPRT in Group B and a known or likely genetic cause (e.g. positive family history of RCC) were diagnosed with their first RCC at a younger age than those without an apparent genetic cause (43.9 years versus 57.1 years (*p* < 2.2 × 10^−16^)) and the male:female predominance was reduced (M:F ratio 1.72 versus 2.47 (*p* > 0.05)). Among all patients with MPRT in Groups A and B and a specified genetic cause (*n* = 469), the most common reported causes were VHL disease (69.7%), Birt-Hogg-Dubé (BHD) syndrome (14.2%), HPRC (*MET* variants) (4.7%), tuberous sclerosis complex (TSC) (6.5%), hereditary leiomyomatosis and renal cell cancer syndrome (HLRCC) (2.4%), succinate dehydrogenase (SDH) deficiency (1.5%) and a constitutional translocation (1.1%). However, these estimates may be biased since some studies only focused on patients with VHL, BHD, HPRC or HLRCC (Supplementary Tables 1 and 2).

In patients with MPRT the tumours might be of the same or different histology (though as CRCC accounts for >70% of RCC by chance it might be predicted that around 50% of patients with MPRT would have multiple CRCC). However, in patients with individual level data (Group B with RCC histology available (*n* = 492)) 79.1% (389/492) of patients with MPRT had tumours of the same histology and 39.6% (195/492) of the total had multiple CRCC, 16.1% (79/492) had multiple PRCC and 7.3% (36/492) multiple ChRCC. Therefore, the frequency of patients with multiple PRCC and ChRCC was higher than expected (compared to a random distribution of histologies). Among patients with MPRT and a diagnosed hereditary cancer syndrome, multiple CRCC was reported in patients with VHL disease (93.5% (43/46)) and constitutional chromosome 3 translocations (100% (5/5) and multiple PRCC in those with germline *MET* protooncogene mutations (95.2% (20/21)). After excluding patients with germline *MET* mutations the frequency of patients with MPRT and multiple PRCC (12.5% (59/471)) was still higher than would be predicted by chance. However, for patients diagnosed with BHD syndrome and TSC the histological concordance was lower (84.3% and 81.0% respectively).

## Discussion

### MPRT and inherited predisposition to RCC

MPRT has often been taken as a clinical indicator of a possible underlying hereditary cancer predisposition and the literature review confirmed the greater frequency (14.9% (430/2879)) of patients with a family history of cancer or a diagnosis of a hereditary RCC-associated syndrome in MPRT compared to an unselected series of patients with RCC (~5%) [17, 18]. However, a potential limitation of analysing cases reported in the literature is preferential reporting of patient series for specific cancer syndromes (e.g. VHL disease). A wide variety of inherited disorders can predispose to RCC (and often other tumour types), and these are summarised in Table 1. The frequency of an inherited RCC predisposition disorder among a cohort with MPRT will depend on multiple factors including the frequency of the inherited disorder, the risk of RCC and MPRT in the disorder and the ease with which it can be diagnosed, for example if there is a readily apparent or distinctive phenotype or because genetic testing for the disorder is widely utilised.

**Table 1:** The genetics and clinical characteristics of autosomal dominant hereditary renal cell carcinoma (RCC)-associated disorders that can be associated with MPRT.

Hereditary RCC-associated syndrome	Genetic cause	RCC histopathology	Major extra-renal features	Risk of RCC	Frequency of multicentric or bilateral RCC
Von Hippel-Lindau (VHL) disease	*VHL* gene	Clear cell RCC and renal cysts	Retinal and cerebellar hemangioblastomas, paraganglioma, pheochromocytoma, pancreatic cyst and neuroendocrine tumours, epididymal cysts, endolymphatic sac tumours	70% [19]	44% [59]
Birt-Hogg- Dubé (BHD) syndrome	*FLCN* gene	Hybrid chromophobe and oncocytic RCC	Fibrofolliculomas, trichodiscomas, pulmonary cysts, pneumothorax	25–30% [60, 61]	18–60% [62, 63]
Hereditary leiomyomatosis and RCC syndrome	*FH* gene	Type 2 papillary RCC, collecting duct RCC	Cutaneous leiomyomas, uterine leiomyomas	21% [24]	4% [24]
Succinate dehydrogenase (SDH) deficiency	*SDHA/B* */C/D* genes	SDH- deficient RCC	Head and neck paragangliomas, extra-adrenal sympathetic paragangliomas, wild-type gastrointestinal stromal tumours, pituitary tumours	<10% [64, 65]	11% [66]
Hereditary papillary RCC	*MET* gene	Type 1 Papillary RCC	/	54% [67]	47% [68]
Chromosome 3 translocation	Chromo some 3	Clear cell RCC	/	Up to 70% [69]	/
Tuberous sclerosis complex	*TSC1* and *TSC2* genes	RCC, angiomyolipoma, oncocytoma	Learning disability, cutaneous fibromas and other skin lesions, subependymal giant cell astrocytoma, hamartomas, pulmonary lymphangioleiomyomatosis, cardiac rhabdomyomas	4% [70]	/
*BAP1* tumour predisposition syndrome	*BAP1* gene	RCC	Uveal and cutaneous melanoma, mesothelioma	10% [71, 72]	/
Cowden syndrome	*PTEN* gene	RCC	Breast, endometrium and follicular thyroid cancer, hamartoma, macrocephaly, mucocutaneous papules/keratosis, facial trichilemmomas	4–35% [73]	11% [74]

Estimates of the percentage of multicentric or bilateral RCC among individuals with RCC in different hereditary RCC-associated syndromes are also cited (Details of references 59–74 are provided in the Supplementary Material).

In this literature review VHL disease was the most frequently reported inherited renal cancer disorder (IRCD) followed by BHD syndrome, HPRC caused by germline MET mutations and TSC. The frequency of VHL disease reflects the high risk of RCC and the prevalence of bilateral/multicentric tumours in VHL disease [19]. Indeed, careful examination of apparently normal renal tissue in VHL disease can reveal hundreds of microscopic early tumour lesions [20]. Furthermore, the onset of RCC in VHL disease is, on average, later than for other major manifestations of VHL disease (e.g. retinal and CNS haemangioblastoma and phaeochromocytoma) and so in patients with isolated non-syndromic MPRT the frequency of VHL disease is lower than might be expected. A predisposition to MPRT is also a well-recognised feature of BHD syndrome though the lifetime risk of RCC is substantially lower than in VHL disease (Table 1). The other major manifestations of BHD syndrome (fibrofolliculomas, pneumothorax and pulmonary cysts) present, on average, at a younger age than RCC (median age ~50 years) but extrarenal features may be overlooked (e.g., fibrofolliculomas) or their significance may not be recognised (e.g., pneumothorax) so that genetic testing of patients with isolated non-syndromic MPRT can reveal a subset with previously undiagnosed BHD syndrome [21].

Tumour histopathology can be a useful indicator of a particular genetic cause of MPRT. As observed in the literature review results, RCC in VHL disease is typically clear cell type whereas HPRC is type 1 papillary RCC and though a variety of tumour types can be seen in BHD syndrome the occurrence of hybrid chromophobe/oncocytic RCC is suggestive of the disorder. MPRT is a well-recognised feature of HPRC, and the population frequency of HPRC which is caused by germline MET mutations is estimated to be <1 per million [22, 23]. RCC in HLRCC is usually classified as type 2 papillary RCC or collecting duct RCC but this usually presents as a unilateral lesion and the frequency of HLRCC as a cause of MPRT is perhaps lower than expected. This probably reflects the lower risk of RCC in HLRCC compared to VHL disease and BHD syndrome and, also, the frequent poor prognosis of the aggressive RCC that are associated with HLRCC [24]. Succinate dehydrogenase deficient (dSDH) RCC associated with germline mutations in SDH subunit genes (*SDHx*) is now a recognised histological subtype of RCC [25]. Suggestive histopathological characteristics of dSDH tumours have been defined but loss of

SDHB immunostaining is an important diagnostic feature of dSDH-RCC [25, 26]. Germline mutations in any of the four genes that encode SDH subunits (*SDHA, SDHB, SDHC, SDHD*) may be associated with dSDH tumour types (e.g. paraganglioma, phaeochromocytoma, head and neck paraganglioma, RCC and wild-type gastrointestinal stromal tumours (GIST)) but RCC has been particularly associated with germline *SDHB* mutations (though the lifetime risk of RCC is likely less than 10%) [27, 28]. To date *SDHx* variants have not been reported as a major cause of MPRT but some studies suggest that their role in genetic predisposition to RCC may have been underestimated [18].

RCC is recognised as a rare feature of TSC, with benign angiomyolipoma representing the majority of renal masses in TSC patients. Non-renal manifestations of TSC include learning disability, epilepsy, cerebral cortical tubers, subependymal astrocytoma, pulmonary lymphangioleiomyomatosis, retinal hamartomas, cutaneous angiofibroma, periungual fibromas, hypopigmented patches and cardiac rhabdomyomas [29]. If multiple RCC occurs it is usually in the presence of other features of TSC so that it is an unlikely cause of non-syndromic MPRT. Cowden syndrome is a multisystem familial cancer syndrome caused by germline mutations in the *PTEN* tumour suppressor gene characterised by breast and endometrial cancers, thyroid and colorectal neoplasia, skin and mucous membrane lesions and macrocephaly. Though the risk of RCC has been reported to be around 30% the frequency in non-syndromic RCC appears to be very low [18, 30].

While there is variability in the gene content of genetic testing panels for MPRT, *VHL, FH, FLCN, MET, SDHB* and *BAP1* are generally included. In addition to predisposing to RCC, germline *BAP1* mutations predispose to mesothelioma (pleural and peritoneal), uveal melanoma and cutaneous melanoma, so the personal or family history of these features should lead to the suspicion of BAP1 tumour predisposition syndrome (BPTS) [31]. Multifocal and bilateral RCC have been reported in BPTS. Inherited renal cancer disorders that should be considered in patients with MPRT suspected of having a genetic cause but who test negative for a standard RCC gene panel test, include germline *SDHx* (*SDHA, SDHC* and *SDHD* if only *SDHB* has been tested), *CHEK2* variants and two recently described RCC predisposition genes (*ELOC* and *PRDM10*) which are associated with syndromic forms of RCC. A de novo missense variant (c.236A > G (p.Tyr79Cys)) in the pVHL-binding protein elongin C (*ELOC* gene) was reported in a de novo case of VHL disease (without a germline *VHL* mutation). The missense variant occurs at a residue that is a hotspot for somatic mutations in a specific form of RCC and for which the mutant ELOC protein has been shown to mimic the cellular effects of a *VHL* mutation [32]. Recently, two independent reports have described the detection of missense variants at the same residue of *PRDM10* gene (c.2029T > C (p.Cys677Arg) and c.2030G > A (p.Cys677Tyr)) in two families with a BHD-like phenotype (including fibrofolliculomas, trichodiscomas, lipomas, lung cyst(s), and RCC) [33, 34]. Two cases of MPRT were reported in the two families. Whilst these findings require confirmation in additional reports, they might be considered for further testing on a research basis in selected cases. Germline mutations in *CHEK2* have been primarily associated with predisposition to breast, prostate and colorectal cancers but there is increasing evidence for an association with RCC with an approximately two-fold increased lifetime risk [35].

Multiple other genes have been reported to potentially predispose to RCC, including *PBRM1, BRIP1, CDKN2B, DCLRE1B/Apollo, MITF* and *NBR1*, but are not routinely tested in clinical practice as they appear to be rare causes of inherited RCC/MPRT and/or require additional confirmation of RCC risks [36–40]. Several genes listed in Table 1, such as *VHL, SDHx* and *FH* are associated with predisposition (to varying extents) to both RCC and PPGL. Two further phaeochromocytoma/paraganglioma (PPGL) predisposition genes, *TMEM127* and *MAX*, have also been linked with RCC predisposition and, whilst not routinely tested for, might be investigated if they are in a patient with MPRT and a personal or family history of PPGL without evidence of a germline mutation on routine testing [41].

Hence, there has been increasing interest in whether routine genetic testing of patients with unselected RCC and with MPRT (irrespective of the presence/absence of renal or extra-renal features of inherited cancer syndrome) might provide a better estimate of the genetic contribution to unselected RCC cases and to MPRT. For example, in an unselected series of 1336 patients with RCC who underwent germline whole genome sequencing the overall detection of pathogenic/likely pathogenic germline variants (PGV aka mutation) in a large panel of cancer susceptibility genes (CSGs) was 6.4% [18]. However not all the CSGs harbouring a PGV were known to be associated with predisposition to RCC (only 4.5% of all cases had a PGVs in a CSG (*VHL, FLCN, MET, TSC1/2, FH, SDHA/B/C/D, BAP1* and *CHEK2*) recognised as being associated with RCC predisposition) [18]. Other studies have reported a similar overall frequency of PGVs in RCC CSGs [17, 42]; and higher frequencies (~9%) have been reported in case series in which ascertainment has been biased towards early onset and/or advanced stage RCC [43, 44]. An extensive genomic analysis focusing on MPRT has not yet been performed within published series of unselected RCC but estimates of the diagnostic yield in MPRT have varied between 7% and 29%, [44, 45]. Recently a number of novel candidate RCC CSGs have been reported that should be considered as potential candidate genes for MPRT in patients without a detectable PGV in a CSG (see above and below).

### Approaches to genetic testing in cases of MPRT with a suspected underlying genetic cause

The presence of extra-renal neoplastic and non-neoplastic clinical features associated with a specific inherited RCC syndrome, both in the patient and their family history, might suggest the likelihood of a specific genetic diagnosis (Table 1). For example, the presence of a retinal or central nervous system haemangioblastoma would raise concerns of VHL disease and pneumothorax could imply BHD syndrome whereas phaeochromocytoma or paraganglioma in combination with RCC might indicate multiple candidate disorders (Table 1) [19, 41]. As described above, renal features such as RCC histopathology can provide clues to likelihood/non-likelihood of certain inherited cancer syndromes. For example, MPRT in VHL disease is almost exclusively clear cell whereas type 1 papillary RCC is seen in HPRC (Table 1) and histopathological analysis can be supplemented by immunohistochemistry to identify specific characteristics of a cancer syndrome such as loss of FH expression and overexpression of 2-SC in HLRCC and loss of SDHB immunostaining in SDH-deficient RCC [26].

Although extra-renal and renal features of specific IRCDs can raise the suspicion of a particular diagnosis (Table 1), many of these disorders have incomplete or age-dependent penetrance and can also present in a non-syndromic form (i.e. with RCC but no extra renal features). Hence, first-line testing for a genetic cause of MPRT is most often performed for a panel of RCC susceptibility genes rather than for a single gene. The number of genes within a panel may be as few as six (e.g. *BAP1, FH, FLCN, MET, SDHB*, and *VHL* genes) though commercial genetic testing companies may offer larger gene panels (18–30 genes but including some for non-RCC kidney tumours; (Supplementary Table 3)). Whilst larger gene panels will increase the number of gene variants detected, this will often be accompanied by more variants of uncertain significance (VUS) and/or genetic variants in cancer predisposition genes that might not be unequivocally linked to the MPRT phenotype. In our experience it is generally reasonable to adopt a two-stage approach to genetic testing in MPRT in patients without syndromic features by starting with a core gene panel of 6–11 genes (*VHL, FLCN, MET, FH, BAP1* and *SDHB* + *CHEK2, PTEN, SDHA, SDHC* and *SDHD*) and cytogenetic analysis for a constitutional translocation. A second round of testing can then be performed for rarer causes of inherited predisposition to RCC in patients considered to be at higher risk of having a genetic cause because of a positive family history and/or younger age at onset. Little information is available on the risks of RCC in first degree relatives of individuals with sporadic/non-syndromic MPRT and negative genetc testing and therefore approaches to offering surveillance to family members are variable. For cases with a relatively young age at onset (<55 years) a pragmatic approach might be to offer a “one-off” renal ultrasound scan to siblings and parents and two-yearly renal ultrasonography (commencing at 5 years before the date of the first cancer in the proband) to the children of the proband. If such an approach is adopted, then surveillance outcomes should be collected on a prospective basis to provide a basis more the development of evidence-based surveillance protocols.

The detection of a germline pathogenic variant in a RCC CSG in a patient with MPRT has important implications for genetic risks of RCC and other tumours both in the patient and in their relatives and enables predictive testing to be offered to family members at risk.

### Potential causes of MPRT in the absence of a detectable genetic predisposition

If first line genetic testing for MPRT provides a negative result, the requesting clinician should re-evaluate the available evidence and decide whether further genetic testing is merited or whether there are other potential explanations for MPRT. If there is still a clinical suspicion of an underlying genetic cause, then potential explanations for negative testing include:

#### False negative gene panel testing result

While genetic testing using gene panels and next generation sequencing testing approaches are generally considered to have high sensitivity for most PGVs, are missense variants might be misclassified as a VUS if there is insufficient evidence for pathogenicity or certain genetic alterations (e.g. structural variants, intronic and regulatory region mutations) might not be detected by targeted sequencing techniques that focus on exonic regions. Constitutional translocations involving chromosome 3 may be associated with predisposition to RCC and can be detected by cytogenetic analysis [46]. Mosaicism may also result in false negative testing results and, if suspected can be confirmed by deep sequencing assays or analysis of DNA from non-blood tissues (including normal renal tissue). In individuals with MPRT who are mosaic for a CSG variant, the presence of an identical PGV in all of the tumours can indicate the underlying genetic cause even if the PGV is not detectable in germline (blood) testing. We note that in a subgroup of children with Wilms tumour somatic epigenetic alterations can be detected in multiple normal kidney tissue samples or renal tumours but not in blood [47]. Interestingly, Merino et al. [48] reported an adult male with early-onset multiple unilateral kidney tumours in which an *IDH2* gain-of-function mutation was detected in the multifocal tumours but not in normal renal tissue. Though this finding raised the possibility that MPRT was being mimicked by metastatic disease, all tumours were small (diameter of 0.6–1.4 cm), distributed throughout the kidney without evidence of localised spread suggesting that the tumours were derived from an abnormal clone of *IDH2* mutated cells in the normal renal parenchyma [48].

#### The relevant CSG has not been tested

Comprehensive genomic sequencing studies have demonstrated that about 2% of patients with RCC have a PGV in a known CSG that is not currently designated as an RCC CSG (e.g. *BRCA1, ATM, BRIP1, TP53*). Further investigations are required to determine if these findings are coincidental or if there is a causal association. Over time RCC may become recognised as a complication of some CSGs that primarily predispose to other phenotypes, for example, recent reports have suggested that *CHEK2* mutations (primarily associated with predisposition to breast, prostate and colorectal cancers) are associated with a two-fold increased risk of RCC [35]. Several candidate RCC CSGs have been described that require further validation before they can be considered suitable for clinical diagnostic testing (see above). In addition, not all genetic factors may operate as rare high penetrance predisposition alleles. In breast and colorectal cancers there is increasing interest in the clinical application of polygenic risk score (PRS) to estimate inherited risks [49]. However, currently GWAS studies have identified relatively few risk alleles for RCC and PRS have yet to be developed and evaluated [49].

#### Misdiagnosis of intrarenal metastatic disease as MPRT

Recently tumour sequencing studies have demonstrated that some patients presenting with apparent MPRT do in fact have intrarenal metastases from a single primary RCC [14]. Whilst this knowledge has major implications for patient management it seems likely that the frequency of this phenomenon will have been underestimated as tumour sequencing studies are not widely available. Factors that would make this possibility more likely would be the presence of extrarenal metastases, an absence of genetic risk factors, identical histopathology in all the tumours and at least one large tumour (>4 cm).

#### Occupational exposure

Carcinogens proposed to be linked to RCC include trichloroethylene (with odds ratio = 1.63 for ever trichloroethylene exposure to never exposure) [50], aristolochic acid (associated with the A:T > T:A mutation signature in RCC) [51] and vitamin A and E synthesis workers [52]. Patients with end-stage renal failure treated with long-term dialysis or transplantation are at risk of developing RCC in non-functioning kidneys [53]. Whilst epidemiological studies have linked RCC risk to obesity, smoking and hypertension [54, 55], to our knowledge, there have been no large-scale studies which have investigated whether these factors are more common in patients with MPRT but it could be postulated that these influences might interact with genetic susceptibility alleles.

### Clinical management of patients with MPRT

**Fig. 2:**
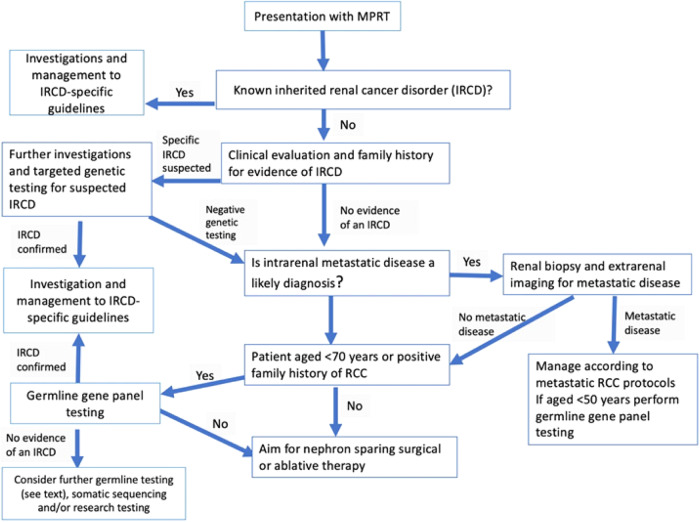
Flow chart outlining steps in the investigation of multiple primary renal tumours (MPRT). IRCD inherited renal cancer disorder, RCC renal cell carcinoma.

Following the diagnosis of MPRT a careful review of the clinical and imaging data is important to determine whether the diagnosis of MPRT is likely to be correct or if the appearance of MPRT might in fact be caused by metastatic disease from a single primary RCC. In most cases, metastatic disease will be unlikely if the largest renal tumour is smaller than 3 cm maximum diameter [56]. However, there may be exceptions to this observation. For example, the aggressive RCC associated with HLRCC can metastasise when smaller than 3 cm whereas in other familial cancer syndromes such as VHL disease, BHD syndrome and HPRC-MET renal tumours are routinely kept under observation until they reach 3 cm diameter and nephron-sparing surgery is performed. If a patient is known to have an inherited cancer syndrome such as VHL disease or BHD syndrome, solid renal lesions are not routinely biopsied because of the high prior probability of an RCC. However, in patients without a known genetic cause, image-guided biopsy may be performed to confirm the diagnosis of RCC or benign lesions (e.g. oncocytoma). The histological and immunohistochemical analysis of the biopsy material can indicate which inherited cancer syndromes are more or less likely (see above and Fig. 2) and the finding of different histologies will make metastatic disease less likely. The gold standard diagnostic test for excluding the possibility of intrarenal metastatic disease phenocopying MPRT would be tumour sequencing and analysis for shared somatic mutations, but this is not widely available. Therefore, an assessment of the likelihood, or not, of metastatic disease will be based on careful consideration of the clinical picture, pathological findings, and absence/presence of metastatic disease on extrarenal imaging of the lungs, abdomen, bones, and brain. The approach to genetic testing is guided by the clinical history and examination, family history and histopathological findings. If these suggest a specific IRCD, then targeted testing can be performed. However, in the absence of a clear clinical diagnosis the clinician will request gene panel testing. If this proves negative and an underlying genetic cause is still suspected then reasons for a false negative genetic testing result should be considered (see above) and options for further testing can be pursued (e.g. large gene panel, genome sequencing, somatic sequencing, immunohistochemical investigations etc.).

Assuming that there is no evidence of metastatic disease, the management of MPRT is to aim for curative intervention whilst preserving non-cancerous functioning renal tissue. This approach of nephron sparing surgery was pioneered for patients with VHL disease and MPRT but has been extended to most (but not all) familial RCC cancer syndromes and sporadic patients. For familial patients under active follow-up MPRT may be detected when individual tumours are small, and intervention delayed until the largest tumour reaches 3 cm in diameter. In such cases the favoured approach has been to remove the largest tumours by partial nephrectomy of the relevant kidney. At the same time, smaller tumours that are easily accessible may be “shelled out”. If there are contralateral tumours that are ~3 cm these can then be treated in sequential manner with staged surgical procedures. Following surgery, any remaining tumours are then kept under surveillance until they reach 3 cm diameter and partial nephrectomy is performed [57]. In some centres less invasive ablative procedures (e.g. radiofrequency ablation) may be offered but it may be more difficult to limit the effect in surrounding normal tissue compared with surgical approaches [58]. However, there are exceptions to this “3 cm guideline” for surgical intervention, for example, in patients with HLRCC, tumours are generally treated when diagnosed and not kept under surveillance and such an approach has also been suggested for renal tumours in patients with germline *BAP1* and *SDHB* mutations. In patients with other major health issues or, multisystem manifestations of an inherited RCC syndrome, surgery may be deferred, and systemic therapy will be instigated (e.g. in patients with VHL disease and CNS haemangioblastoma and/or pancreatic tumours then belzutifan therapy would offer the possibility of treating a variety of tumours without multiple surgeries).

### Future prospects

Currently the investigation and management of MPRT offers multiple examples of the importance of diligent clinical assessment, laboratory investigations and imaging to enable a personalised approach to patient management. The identification of individuals with specific inherited RCC syndromes is critical as those with an underlying genetic cause will likely be at increased risk of further renal primaries and, in many cases, extrarenal tumours requiring surveillance to reduce morbidity and mortality. The approach to the timing of surgery is influenced by the specific IRCD, and options for personalised precision medicine therapies are likely to increase with time. However, current clinical care and genetic testing pathways are likely to result in incomplete identification of cases with an underlying genetic basis and also to those cases of apparent MPRT that are caused by intrarenal metastases. The greater availability of somatic tumour sequencing would greatly facilitate the diagnosis of intrarenal metastases, and the adoption of genome-wide sequencing strategies (e.g. exome and genome sequencing) would enable secondary analysis of a wide range of candidate genes (on a diagnostic or research basis) in individuals with MPRT with negative first-line genetic testing results. The application of immunohistochemistry to detect the products of RCC predisposition genes (e.g. *SDHB*, *FH*/2SC, *BAP1*, etc.) can aid interpretation of equivocal genetic findings, and in vitro and in vivo metabolomic studies may have a role in specific cases. However, there is still considerable variability in approaches to genetic testing in MPRT and the results of systematic clinical and genetic analysis of large cohorts of patients with MPRT would provide a stronger evidence base for developing a consensus on the investigation and management of patients presenting with MPRT without a known or suspected RCC predisposition syndrome.

### Supplementary information

Supplemental Material contents and references

Supplementary figure

Supplementary Table 1

Supplementary Table 2

Supplementary Table 3
