# Supplementary material for: Characteristics, aetiology and implications for management of multiple primary renal tumours: a systematic review
Source: Eur J Hum Genet. 2024 May 27;32(8):887–94. doi: 10.1038/s41431-024-01628-5 (PMC11291654; doi:10.1038/s41431-024-01628-5)
Supplement: Supplementary file 1 — Supplemental Material contents and references [file 41431_2024_1628_MOESM1_ESM.docx]

# SUPPLEMENTARY MATERIAL

# Characteristics, Aetiology and Implications for Management of Multiple Primary Renal Tumours: A Systematic Review

Huairen Zhang^1^, Avgi Andreou^1^, Rupesh Bhatt^2^, James Whitworth^1^, Bryndis Yngvadottir^1^, Eamonn R Maher1,3

1. Department of Medical Genetics, School of Clinical Medicine, University of Cambridge, Cambridge, CB2 0QQ, UK
2. Department of Urology, Queen Elizabeth Hospital, Birmingham B15, UK
3. Aston Medical School, College of Health and Life Sciences, Aston University, Birmingham B4 7ET, UK

**Correspondence to ERM: erm1000@cam.ac.uk or e.maher@aston.ac.uk**

**Contents:**

1. Supplementary References
2. Supplementary Figure 1
3. Supplementary Table 1: The demographic and clinical feature of patients with multiple primary renal tumours in group _A and the histology of the renal tumours.
4. Supplementary Table 2: The demographic and clinical feature of patients with multiple primary renal tumours in group _B and the histology of the renal tumours
5. Supplementary Table 3: Supplementary Table 3. Examples of the renal cell carcinoma (RCC) susceptibility gene panels used for genetic testing

**Supplementary References**

59. Chauveau D, Duvic C, Chrétien Y, Paraf F, Droz D, Melki P, et al. Renal involvement in von Hippel-Lindau disease. Kidney Int. 1996;50(3):944–51.

60. Bruinsma FJ, Dowty JG, Win AK, Goddard LC, Agrawal P, Attina’ D, et al. Update of penetrance estimates in Birt-Hogg-Dubé syndrome. J Med Genet. 2023 Apr;60(4):317–26.

61. Schmidt LS, Nickerson ML, Warren MB, Glenn GM, Toro JR, Merino MJ, et al. Germline BHD-mutation spectrum and phenotype analysis of a large cohort of families with Birt-Hogg-Dubé syndrome. Am J Hum Genet. 2005;

62. Pavlovich CP, Walther MM, Eyler RA, Hewitt SM, Zbar B, Linehan WM, et al. Renal tumors in the Birt-Hogg-Dubé syndrome. Am J Surg Pathol. 2002;

63. Sattler EC, Reithmair M, Steinlein OK. Kidney cancer characteristics and genotype-phenotype-correlations in Birt-Hogg-Dubé syndrome. PLoS One. 2018;13(12):1–8.

64. Christopher J. Ricketts, Brian Shuch, Cathy D. Vocke, Adam R. Metwalli, Gennady Bratslavsky, Lindsay Middelton, Youfeng Yang, Ming-Hui Wei, Stephen E. Pautler, James Peterson, Catherine A. Stolle, Berton Zbar, Maria J. Merino, Laura S. Schmidt PAP. Succinate Dehydrogenase Kidney Cancer (SDH-RCC):Aggressive Example of the Warburg Effect in Cancer. J Urol. 2012;188(6):1–16.

65. Ricketts CJ, Forman JR, Rattenberry E, Bradshaw N, Lalloo F, Izatt L, et al. Tumor risks and genotype-phenotype-proteotype analysis in 358 patients with germline mutations in SDHB and SDHD. Hum Mutat. 2010 Jan;31(1):41–51.

66. Gill AJ, Hes O, Papathomas T, Šedivcová M, Tan PH, Agaimy A, et al. Succinate dehydrogenase (SDH)-deficient renal carcinoma: A morphologically distinct entity: A clinicopathologic series of 36 tumors from 27 patients. Am J Surg Pathol. 2014;38(12):1588–602.

67. Schmidt LS, Nickerson ML, Angeloni D, Glenn GM, Walther MM, Albert PS, et al. Early onset hereditary papillary renal carcinoma: Germline missense mutations in the tyrosine kinase domain of the MET proto-oncogene. J Urol. 2004;172(4 I):1256–61.

68. Lubensky IA, Schmidt L, Zhuang Z, Weirich G, Pack S, Zambrano N, et al. Hereditary and sporadic papillary renal carcinomas with c-met mutations share a distinct morphological phenotype. Am J Pathol. 1999;

69. Smith PS, Whitworth J, West H, Cook J, Gardiner C, Lim DHK, et al. Characterization of renal cell carcinoma-associated constitutional chromosome abnormalities by genome sequencing. Genes Chromosom Cancer. 2020;59(6):333–47.

70. Rakowski SK, Winterkorn EB, Paul E, Steele DJR, Halpern EF, Thiele EA. Renal manifestations of tuberous sclerosis complex: Incidence, prognosis, and predictive factors. Kidney Int [Internet]. 2006;70(10):1777–82. Available from: http://dx.doi.org/10.1038/sj.ki.5001853

71. Popova T, Hebert L, Jacquemin V, Gad S, Caux-Moncoutier V, Dubois-D’Enghien C, et al. Germline BAP1 mutations predispose to renal cell carcinomas. Am J Hum Genet. 2013;92(6):974–80.

72. Rai K, Pilarski R, Cebulla CM, Abdel-Rahman MH. Comprehensive review of BAP1 tumor predisposition syndrome with report of two new cases. Clin Genet. 2016;89(3):285–94.

73. Kim RH, Wang X, Evans AJ, Campbell SC, Nguyen JK, Farncombe KM, et al. Early-onset renal cell carcinoma in PTEN harmatoma tumour syndrome. Vol. 5, NPJ genomic medicine. England; 2020. p. 40.

74. Mester JL, Zhou M, Prescott N EC. Papillary renal cell carcinoma is associated with PTEN hamartoma tumor syndrome. Urology. 2012;79(5).
