# Supplementary material for: Characteristics, aetiology and implications for management of multiple primary renal tumours: a systematic review
Source: Eur J Hum Genet. 2024 May 27;32(8):887–94. doi: 10.1038/s41431-024-01628-5 (PMC11291654; doi:10.1038/s41431-024-01628-5)
Supplement: Supplementary file 2 — Supplementary figure [file 41431_2024_1628_MOESM2_ESM.pptx]

## Slide 1
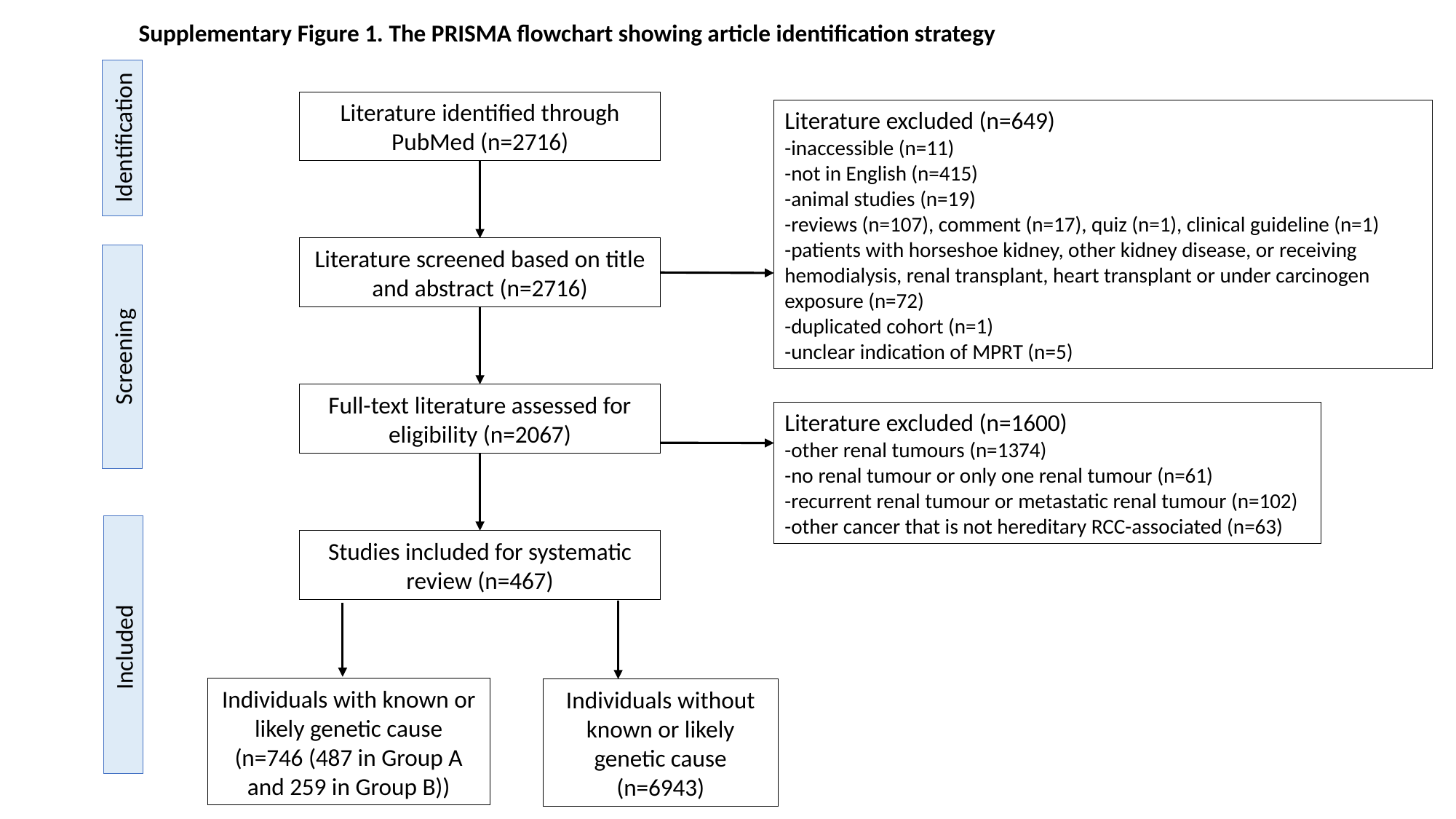

Supplementary Figure 1. The PRISMA flowchart showing article identification strategy
Literature identified through PubMed (n=2716)
Literature excluded (n=649)
-inaccessible (n=11)
-not in English (n=415)
-animal studies (n=19)
-reviews (n=107), comment (n=17), quiz (n=1), clinical guideline (n=1)
-patients with horseshoe kidney, other kidney disease, or receiving hemodialysis, renal transplant, heart transplant or under carcinogen exposure (n=72)
-duplicated cohort (n=1)
-unclear indication of MPRT (n=5)
Identification
Literature screened based on title and abstract (n=2716)
Screening
Full-text literature assessed for eligibility (n=2067)
Literature excluded (n=1600)
-other renal tumours (n=1374)
-no renal tumour or only one renal tumour (n=61)
-recurrent renal tumour or metastatic renal tumour (n=102)
-other cancer that is not hereditary RCC-associated (n=63)
Studies included for systematic review (n=467)
Included
Individuals with known or likely genetic cause (n=746 (487 in Group A and 259 in Group B))
Individuals without known or likely genetic cause (n=6943)
