# Supplementary material for: Characteristics, aetiology and implications for management of multiple primary renal tumours: a systematic review
Source: Eur J Hum Genet. 2024 May 27;32(8):887–94. doi: 10.1038/s41431-024-01628-5 (PMC11291654; doi:10.1038/s41431-024-01628-5)
Supplement: Supplementary file 3 — Supplementary Table 1 [file 41431_2024_1628_MOESM3_ESM.pdf]

Supplementary Table 1. The demographic and clinical feature of patients with multiple primary renal tumours in group _A and the histology of the renal tumours.

			gender			histology												family history		synchronicity		editary RCC-associated syndr	
PMID	N	mean age	male	female	notes	CCRCC	PRCC	ChRCC	other	NOS	hybrid	CCRCC.PRCC	PRCC.ChRCC	CCRCC.ChRCC	CCRCC.other.RCC	PRCC.other.RCC	ChRCC.other.RCC	with.family.hi	without.famil	synchronous	metachronou	syndrome	number of individual with hereditary syndrome
1292075	4	NA	NA	NA	not specified					4								NA	NA	NA	NA	NA	0
1468477	4	NA	NA	NA	metachronous only; bilateral only					4								NA	NA	0	4	NA	0
1949372	8	NA	NA	NA	not specified					8								NA	NA	NA	NA	NA	0
1997691	12	NA	NA	NA	not specified					12								NA	NA	9	3	NA	0
2226589	18	NA	NA	NA	unselected					18								NA	NA	18	0	NA	0
2398558	40	NA	NA	NA	half synchronous half metachronous					40								NA	NA	20	20	NA	0
2926874	56	NA	NA	NA	half synchronous half meta	56												NA	NA	28	28	NA	0
3278131	10	NA	NA	NA	VHL only; bilateral only	9									1			1	NA	10	0	VHL	10
3393775	6	NA	NA	NA	not specified					6								NA	NA	NA	NA	NA	0
7176050	27	61	21	6	bilateral only					27								NA	NA	19	8	NA	0
7438012	61	49	35	26	synchronous only; bilateral only					61								NA	NA	61	0	NA	0
7581531	3	NA	NA	NA	bilateral only					3								NA	NA	NA	NA	NA	0
7637103	11	NA	NA	NA	unselected					11								NA	NA	11	0	NA	0
7853571	16	NA	13	3	unselected	9	4				2				1			NA	NA	16	0	NA	0
7933167	56	62	39	17	unselected	44		9			3							NA	NA	56	0	NA	0
8201682	100	NA	NA	NA	non-familial only					100								0	100	47	53	NA	0
8270406	4	NA	NA	NA	metachronous only; bilateral only					4								NA	NA	0	4	NA	0
8326552	21	NA	NA	NA	not specified					21								NA	NA	NA	NA	NA	0
8437247	12	NA	NA	NA	VHL only					12								NA	NA	7	5	VHL	12
8632528	18	NA	NA	NA	unselected					18								6	12	18	0	NA	0
8990136	6	NA	NA	NA	not specified					6								NA	NA	2	NA	NA	0
9199639	24	NA	NA	NA	unselected					24								NA	NA	24	0	NA	0
9554334	13	NA	NA	NA	not specified					13								NA	NA	1	NA	NA	0
9715275	10	NA	NA	NA	MET only; familial only; papillary R		10											10	0	NA	NA	MET	2
9751329	57	NA	NA	NA	VHL only					NA								45	12	NA	NA	VHL	45
9754765	29	NA	23	6	bilateral only	17	5		3	3	1							0	29	19	10	NA	0
9763075	21	NA	NA	NA	bilateral only					21								0	21	7	14	NA	0
10193951	27	62	16	11	clear cell RCC only	27												NA	NA	27	0	NA	0
10365129	20	NA	NA	NA	unselected					20								NA	NA	20	0	NA	0
10379735	138	68	100	38	oncocytoma only				138									NA	NA	14	6	NA	0
10427143	19	NA	NA	NA	unselected	14	5											NA	NA	19	0	NA	0
10504476	9	NA	NA	NA	unselected	4		2						1	2			NA	NA	9	0	NA	0
10604695	69	NA	NA	NA	unselected					69								NA	NA	69	0	NA	0
10738186	36	NA	NA	NA	unselected	17			14	4					1			NA	NA	36	0	NA	0
10807693	4	NA	NA	NA	familial only					4								4	0	NA	NA	NA	0
11011538	16	NA	NA	NA	unselected	2	1		2	9	2							NA	NA	16	0	NA	0
11040850	85	NA	NA	NA	synchronous only; bilateral only					71	14							NA	NA	85	0	NA	0
11598446	21	NA	NA	NA	not specified					21								NA	NA	14	7	NA	0
11981010	155	NA	114	41	metachronous only; bilateral only					155								NA	NA	0	155	NA	0
12429303	12	NA	NA	NA	non-familial only					12								0	12	7	5	NA	0
12441999	19	NA	NA	NA	clear cell RCC only	19												NA	NA	19	0	NA	0
12474528	29	NA	23	6	not specified					29								NA	NA	NA	NA	NA	0
12629342	44	NA	30	14	non-familial only; bilateral	32	12											0	44	44	0	NA	0
12814678	19	56	15	4	unselected	13	6											NA	NA	19	0	NA	0
12913692	118	65	94	24	non-familial only; unilatera	36	38	2				22	1		14	4	1	NA	118	118	0	NA	0
14532769	40	NA	NA	NA	VHL only					40								NA	NA	NA	NA	VHL	40
14713769	4	NA	NA	NA	oncocytoma only				4									0	4	4	0	NA	0
15149747	37	NA	NA	NA	unselected					37								NA	NA	37	0	NA	0
15161115	5	NA	NA	NA	unselected					5								NA	NA	5	0	NA	0
15351571	69	NA	52	17	non-familial only	40	29											0	69	69	0	NA	0
15841782	16	NA	NA	NA	bilateral only					16								NA	NA	NA	NA	NA	0
16038693	12	NA	5	7	TSC only					12								NA	NA	NA	NA	TSC	12
16104919	18	NA	NA	NA	synchronous only; bilateral only					18								NA	NA	18	0	NA	0
16243792	21	60	18	3	non-familial only; papillary RCC onl		21											0	21	21	0	NA	0
16434318	3	NA	NA	NA	MET only; familial only; papillary R		3											3	0	3	0	MET	3
16890647	31	NA	NA	NA	unselected	20	11											NA	NA	31	0	NA	0
17066264	75	NA	NA	NA	not specified					75								NA	NA	NA	NA	NA	0
17433034	118	60	92	26	synchronous only; bilatera	86	19	5				7	1					11	107	118	0	NA	0
17509291	120	NA	84	36	metachronous only; bilater	107	6			2		5						10	60	0	120	NA	0
17552949	57	62	43	14	non-familial only; synchron	28	12	4	4	9								0	57	57	0	NA	0
17868730	37	67	28	9	non-familial only	1			2	29					4		1	0	37	37	0	NA	0
18454793	140	NA	109	31	non-familial only; synchron	45	40	2		1		25	1	2	19	4	1	0	140	140	0	NA	0
18485459	87	NA	NA	NA	bilateral only	129	40	3										NA	NA	36	51	NA	0
18487007	192	NA	NA	NA	non-familial only; bilateral	102	32					15	1	2	1			0	192	92	100	NA	0
18640001	34	NA	NA	NA	synchronous only; unilater	18	4	1				2		2	3	3	1	NA	NA	34	0	NA	0
18645615	60	NA	49	11	non-familial only	26	23	1				8	1	1				0	60	60	0	NA	0
18774473	10	NA	NA	NA	non-familial only					10								0	10	10	0	NA	0
18782311	69	NA	NA	NA	not specified	45	24											NA	NA	69	0	NA	0
18794106	22	NA	NA	NA	BHD only; familial only					22								22	0	NA	NA	BHD	3

18813125	14	NA	NA	NA	not specified					14								NA	NA	12	NA	NA	0
19088024	26	59	16	10	clear cell RCC only	26												NA	NA	21	5	NA	0
19222893	26	66	19	7	synchronous only; bilateral only													NA	NA	26	0	NA	0
19293973	4	NA	NA	NA	VHL only; familial only	4												4	0	NA	NA	VHL	4
19386349	17	NA	NA	NA	oncocytoma only				17									NA	NA	17	0	NA	0
19440022	15	NA	13	2	unselected	10	3	1		1								NA	NA	15	0	NA	0
19526183	3	NA	NA	NA	unselected				3									NA	NA	NA	NA	NA	0
19597028	198	59	123	76	bilateral only					198								NA	NA	86	112	NA	0
19751262	73	NA	64	9	non-familial only; synchror	76	41	7	15	4								0	73	73	0	NA	0
19922319	6	NA	NA	NA	non-familial only; synchronous only; unilateral only							3	1	1	1			0	6	6	0	NA	0
19953334	165	NA	NA	NA	non-familial only	147	11	4			3							0	165	NA	NA	NA	0
20299060	9	NA	NA	NA	VHL only; familial only													9	0	NA	NA	VHL	9
20346577	44	61	37	7	unselected	17	22	3	2									3	34	44	0	NA	0
20367634	14	NA	NA	NA	unselected					14								NA	NA	14	0	NA	0
20398456	3	NA	NA	NA	bilateral only; oncocytoma only					3								NA	NA	NA	NA	NA	0
20459372	5	NA	NA	NA	unselected					5								NA	NA	5	0	NA	0
20478582	58	44	22	36	familial only	44	7	5	1		3							58	0	58	0	NA	0
20482658	53	58	38	15	non-familial only; bilateral	47		1		4					1			0	53	31	22	NA	0
20553256	64	NA	NA	NA	clear cell RCC only	64												5	59	NA	NA	NA	0
21107560	2	NA	NA	NA	unselected					2								NA	NA	NA	NA	NA	0
21136181	36	NA	NA	NA	non-familial only					36								0	36	36	0	NA	0
21166703	17	NA	NA	NA	unselected					17								NA	NA	17	0	NA	0
21166765	17	NA	NA	NA	metachronous only				8	5					3	1		NA	NA	0	17	NA	0
21199286	8	NA	NA	NA	non-familial only					8								0	8	NA	NA	NA	0
21239013	17	NA	NA	NA	not specified					17								NA	NA	15	2	NA	0
21496834	40	57	30	10	23 with BHD and 17 without BHD; bilateral only				25	15								0	40	NA	NA	BHD	23
21496838	89	NA	NA	NA	familial only					89								89	0	64	25	NA	0
21611108	11	NA	NA	NA	clear cell RCC only	11												NA	NA	NA	NA	NA	0
21855967	26	56	22	4	synchronous only; bilatera	28	12		5	5								NA	NA	26	0	NA	0
21937095	290	NA	NA	NA	non-familial only; synchror	132	46	3	41	4		23	5	9	19	1	7	NA	290	290	0	NA	0
22018152	16	NA	NA	NA	unselected					16								NA	NA	NA	NA	NA	0
22099962	5	NA	NA	NA	unselected					5								NA	NA	5	0	NA	0
22305422	10	NA	NA	NA	unselected					10								NA	NA	10	0	NA	0
22426863	29	NA	NA	NA	VHL only					29								NA	NA	NA	NA	VHL	29
22441339	334	NA	NA	NA	unselected					334								NA	NA	0	334	NA	0
22502873	249	62	175	74	no hereditary RCC syndron	172	59	9	2	7								NA	NA	249	0	no	0
22959191	5	NA	NA	NA	unselected					5								0	5	5	0	NA	0
23597159	2	NA	NA	NA	unselected					2								NA	NA	NA	NA	NA	0
23619828	3	NA	NA	NA	unselected	3												NA	NA	NA	NA	NA	0
23953228	3	NA	NA	NA	chromophobe RCC only			3										NA	NA	3	0	NA	0
24053513	29	NA	NA	NA	non-familial only					29								0	29	NA	NA	NA	0
24413778	5	NA	NA	NA	VHL only					5								NA	NA	5	0	VHL	5
25391617	12	NA	NA	NA	VHL only	12												NA	NA	12	0	VHL	12
25579143	2	NA	NA	NA	unselected					2								NA	NA	NA	NA	NA	0
26291563	28	NA	NA	NA	unselected					28								NA	NA	NA	NA	NA	0
26861062	18	NA	NA	NA	unselected					18								NA	NA	8	10	NA	0
27136191	60	NA	48	12	non-familial only; synchror	107	7	3	3									0	60	60	0	NA	0
27379623	65	NA	NA	NA	non-familial only; bilateral	110	6	9	2									0	65	36	29	NA	0
28011501	18	NA	NA	NA	unselected					18								NA	NA	18	0	NA	0
28081704	12	NA	10	2	synchronous only; bilateral only; oncocytoma onl				12									NA	NA	12	0	NA	0
28245846	32	55	28	4	non-familial only; synchror	57	5	2										0	32	32	0	NA	0
28469302	12	NA	NA	NA	unselected					12								NA	NA	NA	NA	NA	0
28648756	148	NA	117	31	non-familial only; bilateral	297	9	4	4	3								0	148	88	60	NA	0
28678572	23	NA	16	7	non-familial only	32	11	4		2								0	23	23	0	NA	0
28866246	2	NA	NA	NA	VHL only					2								NA	NA	NA	NA	VHL	2
29370814	27	NA	22	5	synchronous only					1					23	2	1	NA	NA	27	0	NA	0
29733801	5	NA	NA	NA	pediatric RCC					5								NA	NA	NA	NA	NA	0
29897139	21	NA	NA	NA	unselected	4	12	2	3									0	21	21	0	NA	0
30443869	32	57	23	9	non-familial only	14	15	1	2									0	32	32	0	NA	0
30561791	1063	NA	739	324	metachronous only; bilater	900	109	15	39									NA	NA	0	1063	NA	0
30741849	138	74	96	42	bilateral only	89	9	2	11									NA	NA	NA	NA	NA	0
30745424	107	NA	NA	NA	VHL only					107								NA	NA	NA	NA	VHL	107
30922860	44	56	35	9	no hereditary RCC syndron	29	3		2	3		3		1	2	1		NA	NA	22	22	no	0
31231128	11	NA	NA	NA	chromophobe RCC, oncocytoma and hybı			3	2								6	0	11	NA	NA	NA	0
32515677	8	NA	NA	NA	non-familial only					8								0	8	0	8	NA	0
32774235	3	NA	NA	NA	unselected					3								NA	NA	3	0	NA	0
34024273	89	55	74	15	not specified					89								NA	NA	44	45	NA	0
34182558	3	NA	NA	NA	unselected					3								NA	NA	3	0	NA	0
34561376	3	NA	NA	NA	HLRCC only					3								2	1	3	0	HLRCC	3
38267302	18	NA	NA	NA	suspected with hereditary RCC					18								NA	NA	NA	NA	NA	0
37862613	143	NA	NA	NA	unselected					143								NA	NA	NA	NA	NA	0
37555194	7	NA	NA	NA	RCC diagnosed ≤ 46 years old					7								NA	NA	NA	NA	NA	0
