# Supplementary material for: Characteristics, aetiology and implications for management of multiple primary renal tumours: a systematic review
Source: Eur J Hum Genet. 2024 May 27;32(8):887–94. doi: 10.1038/s41431-024-01628-5 (PMC11291654; doi:10.1038/s41431-024-01628-5)
Supplement: Supplementary file 4 — Supplementary Table 2 [file 41431_2024_1628_MOESM4_ESM.pdf]

Supplementary Table 2. The demographic and clinical feature of patients with multiple primary renal tumours in group _B and the histology of the renal tumours.

patient ID	PMID	age	gender	histology	synchronocity	family_history	syndrome	notes
1	22129157	53	male	NOS	synchronous	sporadic	NA	
2	22129157	44	male	NOS	synchronous	sporadic	NA	
3	22129157	35	female	hybrid	synchronous	sporadic	NA	
4	22129157	55	male	NOS	synchronous	sporadic	NA	
5	22129157	65	male	NOS	synchronous	sporadic	NA	non-familial only; synchronous only; bilateral only
6	22129157	42	male	NOS	synchronous	sporadic	NA	
7	22129157	81	male	NOS	synchronous	sporadic	NA	
8	22129157	59	male	NOS	synchronous	sporadic	NA	
9	22129157	42	female	NOS	synchronous	sporadic	NA	
10	22129157	72	male	NOS	synchronous	sporadic	NA	
11	22129157	53	female	NOS	synchronous	sporadic	NA	
12	22129157	61	male	NOS	synchronous	sporadic	NA	
13	24992402	60	male	multiple_PRCC	synchronous	sporadic	NA	
14	32775388	50	male	CCRCC_ChRCC	synchronous	sporadic	NA	
15	27383411	63	male	multiple_CCRCC	synchronous	sporadic	NA	
16	27383411	52	male	multiple_CCRCC	synchronous	sporadic	NA	
17	32914073	62	male	CCRCC_other	synchronous	NA	NA	
18	33564613	78	male	CCRCC_PRCC	synchronous	NA	NA	
19	33564613	76	male	multiple_CCRCC	synchronous	NA	NA	
20	33564613	63	male	multiple_CCRCC	synchronous	NA	NA	
21	31579217	47	male	multiple_CCRCC	synchronous	sporadic	NA	
22	26857533	57	male	CCRCC_PRCC	synchronous	sporadic	NA	
23	16075151	63	NOS	PRCC_other	metachronou	sporadic	NA	
24	16075151	62	NOS	multiple_oncocytoma	synchronous	sporadic	NA	
25	12442000	42	NOS	other	synchronous	sporadic	NA	
26	12442000	62	NOS	multiple_CCRCC	synchronous	sporadic	NA	
27	12442000	71	NOS	multiple_CCRCC	synchronous	sporadic	NA	
28	12442000	58	NOS	multiple_CCRCC	metachronou	sporadic	NA	non-familial only; bilateral only
29	12442000	41	NOS	multiple_CCRCC	metachronou	sporadic	NA	
30	12442000	49	NOS	multiple_CCRCC	metachronou	sporadic	NA	
31	12442000	62	NOS	other	metachronou	sporadic	NA	
32	12442000	54	NOS	multiple_CCRCC	metachronou	sporadic	NA	
33	27158455	68	male	CCRCC_other	synchronous	NA	NA	
34	29255679	56	male	CCRCC_PRCC	synchronous	NA	NA	
35	30671276	68	male	multiple_CCRCC	synchronous	sporadic	NA	
36	23627203	56	male	multiple_CCRCC	synchronous	NA	NA	
37	30774550	52	male	multiple_CCRCC	synchronous	sporadic	NA	
38	28420902	58	male	NOS	synchronous	NA	NA	
39	25180117	67	male	CCRCC_PRCC	synchronous	NA	NA	

40	15816631	70	male	PRCC_other	synchronous	NA	NA	
41	33311425	39	male	CCRCC_ChRCC	synchronous	sporadic	NA	
42	25351926	45	female	hybrid	metachronou	family history	BHD	
43	8689612	58	female	other	synchronous	sporadic	NA	non-familial only; synchronous only; bilateral only
44	8689612	62	male	multiple_CCRCC	synchronous	sporadic	NA	
45	8689612	69	male	multiple_CCRCC	synchronous	sporadic	NA	
46	8689612	68	male	PRCC_other	synchronous	sporadic	NA	
47	8689612	51	male	multiple_PRCC	synchronous	sporadic	NA	
48	25093518	NOS	NOS	other	NA	NA	TSC	
49	25093518	NOS	NOS	other	NA	NA	TSC	
50	25093518	NOS	NOS	other	NA	NA	TSC	
51	25093518	NOS	NOS	other	NA	NA	TSC	
52	26530869	44	male	multiple_CCRCC	synchronous	sporadic	NA	
53	26530869	33	male	multiple_CCRCC	synchronous	sporadic	NA	
54	34153307	10	male	NOS	metachronou	sporadic	TSC	
55	18636147	42	female	multiple_PRCC	NA	sporadic	MET	
56	8371399	65	male	multiple_oncocytoma	synchronous	NA	NA	
57	29866239	60	female	CCRCC_ChRCC	synchronous	NA	NA	
58	23409424	26	female	CCRCC_other	synchronous	sporadic	NA	
59	28620558	55	male	other	synchronous	NA	NA	
60	9041160	NOS	female	multiple_CCRCC	synchronous	NA	NA	unselected
61	9041160	NOS	female	multiple_ChRCC	synchronous	NA	NA	
62	9041160	NOS	male	multiple_ChRCC	synchronous	NA	NA	
63	9041160	NOS	male	multiple_CCRCC	synchronous	NA	NA	
64	9041160	NOS	female	multiple_CCRCC	synchronous	NA	NA	
65	9041160	NOS	male	multiple_ChRCC	synchronous	NA	NA	
66	9041160	NOS	female	multiple_CCRCC	synchronous	NA	NA	
67	9041160	NOS	female	multiple_CCRCC	synchronous	NA	NA	
68	9041160	NOS	male	multiple_PRCC	synchronous	NA	NA	
69	9041160	NOS	male	multiple_CCRCC	synchronous	NA	NA	
70	9041160	NOS	female	multiple_CCRCC	synchronous	NA	NA	
71	9041160	NOS	male	NOS	synchronous	NA	NA	
72	9041160	NOS	male	multiple_CCRCC	synchronous	NA	NA	
73	20108343	7	male	multiple_ChRCC	synchronous	NA	TSC	
74	17211573	40	female	multiple_ChRCC	NOS	NA	BHD	
75	17211573	60	male	multiple_CCRCC	NOS	NA	BHD	
76	17211573	60	female	multiple_CCRCC	NOS	NA	BHD	
77	34447838	56	male	CCRCC_ChRCC	synchronous	sporadic	NA	
78	19997515	48	male	NOS	synchronous	sporadic	NA	non-familial only; bilateral only
79	19997515	54	female	NOS	metachronou	sporadic	NA	
80	19997515	61	male	NOS	metachronou	sporadic	NA	
81	19997515	62	male	NOS	metachronou	sporadic	NA	

82	19997515	42	male	NOS	synchronous	sporadic	NA	
83	26426379	61	male	PRCC_other	synchronous	NA	NA	
84	26426379	49	male	PRCC_other	synchronous	NA	NA	
85	26426379	71	male	other	synchronous	NA	NA	
86	26426379	36	male	other	synchronous	NA	NA	no VHL; include at least one clear cell papillary RCC
87	26426379	68	male	other	synchronous	NA	NA	
88	26426379	65	female	other	synchronous	NA	NA	
89	26426379	64	male	other	synchronous	NA	NA	
90	26426379	60	male	ChRCC_other	synchronous	NA	NA	
91	26426379	72	male	CCRCC_other	synchronous	NA	NA	
92	28009417	38	male	CCRCC_ChRCC	synchronous	NA	BHD	
93	25568749	53	male	multiple_PRCC	synchronous	NA	NA	
94	24134681	39	female	multiple_ChRCC	synchronous	sporadic	NA	
95	11218021	58	male	multiple_PRCC	synchronous	sporadic	NA	
96	10504478	NOS	NOS	CCRCC_PRCC	NA	NA	NA	
97	10504478	NOS	NOS	multiple_PRCC	NA	NA	NA	
98	10504478	NOS	NOS	CCRCC_PRCC	NA	NA	NA	
99	10504478	NOS	NOS	multiple_PRCC	NA	NA	NA	
100	10504478	NOS	NOS	multiple_PRCC	NA	NA	NA	
101	10504478	NOS	NOS	multiple_PRCC	NA	NA	NA	
102	10504478	NOS	NOS	CCRCC_PRCC	NA	NA	NA	
103	10504478	NOS	NOS	multiple_PRCC	NA	NA	NA	
104	10504478	NOS	NOS	multiple_PRCC	NA	NA	NA	
105	10504478	NOS	NOS	multiple_CCRCC	NA	NA	NA	unselected
106	10504478	NOS	NOS	CCRCC_PRCC	NA	NA	NA	
107	10504478	NOS	NOS	multiple_CCRCC	NA	NA	NA	
108	10504478	NOS	NOS	multiple_CCRCC	NA	NA	NA	
109	10504478	NOS	NOS	multiple_CCRCC	NA	NA	NA	
110	10504478	NOS	NOS	multiple_PRCC	NA	NA	NA	
111	10504478	NOS	NOS	CCRCC_PRCC	NA	NA	NA	
112	10504478	NOS	NOS	CCRCC_PRCC	NA	NA	NA	
113	10504478	NOS	NOS	CCRCC_PRCC	NA	NA	NA	
114	7928943	NOS	NOS	NOS	synchronous	NA	NA	
115	27630905	53	male	multiple_PRCC	synchronous	sporadic	NA	
116	29282443	47	female	multiple_CCRCC	synchronous	family history	NA	
117	34121717	45	male	multiple_CCRCC	synchronous	sporadic	NA	
118	19692322	62	female	multiple_CCRCC	metachronou	NA	NA	
119	3617260	54	male	multiple_CCRCC	synchronous	N	NA	
120	24416491	62	male	other			NA	
121	27579386	42	male	multiple_CCRCC	synchronous	sporadic	NA	
122	12195309	0.5	female	multiple_PRCC	synchronous	NA	TSC	
123	3120404	73	female	CCRCC_PRCC	synchronous	NA	NA	

124	17141824	71	female	multiple_CCRCC	metachronou	sporadic	NA	
125	11817296	74	male	multiple_oncocytoma	synchronous	NA	NA	
126	15152522	NOS	NOS	NOS	synchronous	NA	NA	
127	11150957	74	female	multiple_CCRCC	synchronous	NA	NA	
128	28937383	69	male	PRCC_ChRCC	synchronous	sporadic	NA	
129	31779775	36	male	multiple_CCRCC	synchronous	NA	NA	
130	8034424	48	female	NOS	synchronous	NA	NA	
131	9561578	52	male	multiple_CCRCC	metachronou	NA	NA	
132	11586235	71	female	multiple_ChRCC	synchronous	NA	NA	
133	10687967	48	male	NOS	synchronous	NA	NA	
134	10687967	59	male	multiple_oncocytoma	synchronous	NA	NA	
135	10687967	59	male	NOS	synchronous	NA	NA	
136	10687967	68	male	NOS	synchronous	NA	NA	
137	4057415	70	male	PRCC_other	synchronous	NA	NA	
138	16360474	63	male	multiple_oncocytoma	synchronous	NA	VHL	
139	3610137	28	male	PRCC_other	synchronous	sporadic	NA	
140	2224772	53	female	multiple_CCRCC	synchronous	NA	NA	
141	2224772	67	female	multiple_CCRCC	synchronous	NA	NA	
142	2224772	67	male	multiple_CCRCC	synchronous	NA	NA	synchronous only
143	2224772	78	male	multiple_CCRCC	synchronous	NA	NA	
144	2224772	36	female	multiple_CCRCC	synchronous	NA	NA	
145	2224772	40	NOS	multiple_CCRCC	synchronous	NA	NA	
146	2224772	73	male	PRCC_other	synchronous	NA	NA	
147	14666973	75	NOS	PRCC_other	synchronous	NA	NA	
148	18304223	38	female	NOS			TSC	
149	22611408	56	female	CCRCC_other	synchronous	NA	BHD	
150	12547168	74	male	multiple_CCRCC	synchronous	NA	NA	
151	2224628	68	female	multiple_oncocytoma	synchronous	NA	NA	
152	6690725	64	female	multiple_CCRCC	synchronous	NA	NA	
153	18825628	61	male	CCRCC_PRCC	synchronous	NA	NA	
154	8339219	61	male	multiple_PRCC	synchronous	NA	NA	
155	21333329	31	male	multiple_ChRCC	synchronous	NA	NA	
156	26874573	45	male	multiple_CCRCC	synchronous	NA	NA	
157	8183297	36	male	NOS	synchronous	family history	NA	
158	8183297	44	male	NOS	NA	family history	NA	
159	11204268	73	male	CCRCC_other	synchronous	NA	NA	
160	8159926	75	male	multiple_PRCC	synchronous	NA	NA	
161	19261099	57	male	PRCC_ChRCC	synchronous	NA	NA	
162	19616833	59	male	other	NA	NA	NA	
163	19616833	59	male	CCRCC_other	NA	NA	NA	
164	19616833	55	male	multiple_CCRCC	NA	NA	NA	
165	19616833	69	male	multiple_PRCC	NA	NA	NA	

166	32760808	69	male	multiple_CCRCC	synchronous	NA	NA	
167	8592324	35	female	other	synchronous	family history	TSC	
168	8592324	27	female	CCRCC_other	synchronous	family history	TSC	
169	22470760	48	female	CCRCC_ChRCC	synchronous	sporadic	NA	
170	28891800	70	male	hybrid	synchronous	family history	BHD	
171	9507818	44	male	multiple_CCRCC	synchronous	NA	NA	
172	9507818	60	male	multiple_CCRCC	synchronous	NA	NA	
173	9507818	58	female	multiple_CCRCC	synchronous	NA	NA	
174	9507818	57	male	PRCC_other	synchronous	NA	NA	
175	9507818	54	female	multiple_CCRCC	synchronous	NA	NA	unselected
176	9507818	82	male	multiple_CCRCC	synchronous	NA	NA	
177	9507818	53	male	multiple_CCRCC	synchronous	NA	NA	
178	9507818	68	male	multiple_CCRCC	synchronous	NA	NA	
179	9507818	82	female	CCRCC_other	synchronous	NA	NA	
180	9507818	61	female	CCRCC_other	synchronous	NA	NA	
181	3383938	54	female	multiple_CCRCC	synchronous	NA	NA	
182	7448507	37	male	multiple_CCRCC	metachronou	family history	NA	
183	7448507	39	male	multiple_CCRCC	synchronous	family history	NA	
184	7448507	NOS	male	NOS	NA	family history	NA	
185	26170866	42	male	PRCC_other	synchronous	sporadic	NA	
186	17908657	58	male	CCRCC_PRCC	synchronous	NA	NA	
187	3992787	38	male	NOS	synchronous	NA	NA	
188	2769845	58	male	multiple_CCRCC	synchronous	NA	NA	
189	2769845	71	male	NOS	synchronous	NA	NA	
190	2769845	76	male	multiple_CCRCC	synchronous	NA	NA	
191	2769845	36	female	multiple_CCRCC	synchronous	NA	NA	
192	33981609	47	male	CCRCC_other	synchronous	NA	NA	
193	24375046	32	male	multiple_PRCC	synchronous	NA	NA	
194	424835	NOS	NOS	NOS	NA	NA	VHL	
195	12579214	54	male	NOS	synchronous	NA	NA	
196	26613833	56	male	multiple_PRCC	synchronous	NA	NA	
197	26613833	78	female	CCRCC_ChRCC	synchronous	NA	NA	
198	26613833	66	male	NOS	synchronous	NA	NA	
199	26613833	74	female	CCRCC_ChRCC	synchronous	NA	NA	
200	26613833	43	male	multiple_PRCC	synchronous	NA	NA	
201	26613833	63	female	CCRCC_other	synchronous	NA	NA	
202	26613833	50	female	CCRCC_ChRCC	synchronous	NA	NA	synchronous
203	26613833	88	female	NOS	synchronous	NA	NA	only
204	26613833	75	male	CCRCC_PRCC	synchronous	NA	NA	
205	26613833	62	male	CCRCC_other	synchronous	NA	NA	
206	26613833	63	male	CCRCC_PRCC	synchronous	NA	NA	
207	26613833	74	male	CCRCC_PRCC	synchronous	NA	NA	

208	26613833	56	female	CCRCC_PRCC	synchronous	NA	NA	
209	26613833	75	male	CCRCC_PRCC	synchronous	NA	NA	
210	23853020	17	male	CCRCC_ChRCC	synchronous	NA	TSC	
211	20630139	73	male	CCRCC_ChRCC	synchronous	NA	NA	
212	9839601	49	male	NOS	synchronous	NA	chromosomal translocation	
213	9057961	63	male	CCRCC_PRCC	metachronou	NA	NA	
214	22386253	8	female	multiple_CCRCC	synchronous	NA	NA	
215	11551094	58	female	multiple_PRCC	NA	family history	NA	
216	11551094	48	male	CCRCC_PRCC	NA	family history	NA	
217	11551094	55	male	multiple_PRCC	NA	family history	NA	
218	11551094	60	male	multiple_PRCC	NA	family history	NA	
219	11551094	67	male	multiple_PRCC	NA	family history	NA	include at least
220	11551094	68	male	multiple_PRCC	NA	family history	NA	one papillary RCC
221	11551094	71	male	multiple_PRCC	NA	family history	NA	
222	11551094	71	male	multiple_PRCC	NA	sporadic	NA	
223	11551094	47	male	multiple_PRCC	NA	family history	NA	
224	11551094	73	male	CCRCC_PRCC	NA	family history	NA	
225	926269	67	female	NOS	synchronous	NA	NA	
226	926269	37	male	NOS	synchronous	NA	NA	
227	926269	66	male	NOS	synchronous	NA	NA	
228	926269	56	male	NOS	metachronou	NA	NA	
229	926269	56	female	NOS	metachronou	NA	NA	bilateral only
230	926269	61	male	NOS	metachronou	NA	NA	
231	926269	61	male	NOS	metachronou	NA	NA	
232	926269	78	male	NOS	synchronous	NA	NA	
233	926269	64	male	NOS	synchronous	NA	NA	
234	30458744	56	female	multiple_CCRCC	metachronou	sporadic	NA	
235	31726842	69	female	CCRCC_PRCC	synchronous	NA	NA	
236	7029009	52	male	NOS	metachronou	NA	NA	
237	7029009	62	male	NOS	metachronou	NA	NA	
238	7029009	53	male	NOS	synchronous	NA	NA	
239	7029009	71	male	multiple_CCRCC	synchronous	NA	NA	
240	621805	61	male	NOS	synchronous	NA	NA	
241	621805	61	female	NOS	synchronous	NA	NA	
242	621805	49	male	NOS	metachronou	NA	NA	bilateral only
243	621805	52	male	NOS	metachronou	NA	NA	
244	621805	52	male	NOS	metachronou	NA	NA	
245	621805	59	male	NOS	metachronou	NA	NA	
246	10930916	77	male	multiple_PRCC	synchronous	sporadic	NA	
247	10930916	67	female	multiple_PRCC	synchronous	sporadic	NA	
248	10930916	64	male	multiple_PRCC	synchronous	sporadic	NA	

249	10930916	50	female	NOS	metachronou	sporadic	NA	
250	10930916	46	male	NOS	metachronou	sporadic	NA	
251	10930916	54	male	NOS	metachronou	sporadic	NA	non-familial only;
252	10930916	50	male	NOS	synchronous	sporadic	NA	bilateral only
253	10930916	64	male	NOS	synchronous	sporadic	NA	
254	10930916	59	female	NOS	synchronous	sporadic	NA	
255	10930916	79	male	NOS	synchronous	sporadic	NA	
256	10930916	69	female	NOS	synchronous	sporadic	NA	
257	10930916	59	female	NOS	synchronous	sporadic	NA	
258	15018122	62	male	CCRCC_ChRCC	synchronous	sporadic	NA	
259	442378	45	female	NOS	synchronous	NA	NA	
260	33023600	61	male	CCRCC_PRCC	synchronous	NA	NA	
261	21676439	49	male	multiple_CCRCC	synchronous	NA	NA	
262	23865145	42	male	multiple_CCRCC	synchronous	NA	NA	
263	8516223	11	NOS	multiple_CCRCC	synchronous	NA	NA	
264	3787204	63	male	NOS	synchronous	NA	NA	
265	10191351	10	male	multiple_PRCC	synchronous	sporadic	NA	
266	34168986	64	male	multiple_CCRCC	synchronous	sporadic	NA	
267	29887709	34	male	multiple_PRCC	synchronous	NA	NA	
268	9866790	76	female	multiple_CCRCC	synchronous	NA	NA	
269	22369180	67	male	CCRCC_other	synchronous	NA	NA	
270	7225975	NOS	NOS	NOS	NA	NA	NA	
271	30586397	56	female	multiple_ChRCC	NA	family history	BHD	
272	30586397	46	female	multiple_ChRCC	NA	family history	BHD	
273	30586397	NOS	male	NOS	NA	family history	BHD	BHD only;
274	30586397	50	male	multiple_ChRCC	NA	family history	BHD	familial only
275	30586397	69	male	multiple_CCRCC	NA	family history	BHD	
276	30586397	80	male	multiple_CCRCC	NA	family history	BHD	
277	30586397	45	female	CCRCC_other	NA	family history	BHD	
278	3317262	14	female	multiple_CCRCC	synchronous	sporadic	TSC	
279	27732979	59	male	NOS	synchronous	NA	NA	
280	27732979	42	male	NOS	synchronous	NA	NA	
281	27732979	64	male	NOS	synchronous	NA	NA	
282	7887869	62	male	multiple_oncocytoma	synchronous	NA	NA	
283	15287997	70	female	NOS	synchronous	NA	NA	
284	5543057	63	female	multiple_CCRCC	synchronous	NA	NA	
285	24945012	69	male	NOS	synchronous	NA	NA	
286	15471666	71	male	multiple_oncocytoma	synchronous	sporadic	NA	
287	15792133	69	male	multiple_CCRCC	metachronou	sporadic	NA	
288	33616415	38	female	CCRCC_other	synchronous	sporadic	NA	
289	15028029	46	male	multiple_PRCC	synchronous	sporadic	NA	
290	3308788	66	female	NOS	synchronous	NA	NA	

291	3308788	77	male	NOS	metachronou	NA	NA	
292	24136488	46	female	multiple_CCRCC	synchronous	NA	NA	
293	1433089	28	female	NOS	synchronous	NA	NA	
294	1433089	30	female	NOS	synchronous	NA	NA	
295	1433089	63	male	multiple_oncocytoma	synchronous	NA	NA	
296	32022527	43	female	CCRCC_PRCC	synchronous	sporadic	NA	
297	29960980	46	female	multiple_ChRCC	synchronous	family history	TSC	
298	29960980	32	male	multiple_ChRCC	synchronous	family history	TSC	
299	7394964	54	female	NOS	metachronou	NA	NA	
300	16012408	35	female	NOS	synchronous	NA	NA	
301	1743237	42	female	NOS	synchronous	NA	NA	
302	19933033	51	male	multiple_CCRCC	synchronous	NA	NA	
303	10840419	42	female	multiple_CCRCC	synchronous	NA	NA	
304	10840419	58	male	multiple_CCRCC	synchronous	NA	NA	
305	10840419	70	male	multiple_CCRCC	synchronous	NA	NA	
306	10840419	65	male	multiple_CCRCC	synchronous	NA	NA	
307	10840419	64	male	multiple_PRCC	synchronous	NA	NA	
308	10840419	63	male	multiple_CCRCC	synchronous	NA	NA	
309	10840419	49	female	multiple_CCRCC	synchronous	NA	NA	
310	10840419	38	male	multiple_PRCC	synchronous	NA	NA	
311	10840419	61	male	multiple_PRCC	synchronous	NA	NA	
312	10840419	49	male	multiple_CCRCC	synchronous	NA	NA	
313	10840419	65	male	multiple_CCRCC	synchronous	NA	NA	unselected
314	10840419	57	male	multiple_CCRCC	synchronous	NA	NA	
315	10840419	77	male	multiple_PRCC	synchronous	NA	NA	
316	10840419	52	male	multiple_CCRCC	synchronous	NA	NA	
317	10840419	58	male	multiple_CCRCC	synchronous	NA	NA	
318	10840419	53	female	multiple_CCRCC	synchronous	NA	NA	
319	10840419	44	male	multiple_CCRCC	synchronous	NA	NA	
320	10840419	41	female	multiple_PRCC	synchronous	NA	NA	
321	10840419	45	male	multiple_PRCC	synchronous	NA	NA	
322	10840419	67	male	multiple_CCRCC	synchronous	NA	NA	
323	10840419	43	female	multiple_CCRCC	synchronous	NA	NA	
324	10840419	44	male	multiple_CCRCC	synchronous	NA	NA	
325	6492276	63	male	multiple_oncocytoma	NA	NA	NA	
326	6492276	59	male	multiple_oncocytoma	NA	NA	NA	
327	20952280	34	female	NOS	synchronous	NA	VHL	
328	3395987	58	NOS	NOS	NA	sporadic	NA	
329	3395987	71	NOS	NOS	NA	sporadic	NA	
330	3395987	36	NOS	NOS	synchronous	sporadic	NA	
331	3395987	76	NOS	NOS	synchronous	sporadic	NA	
332	29119638	53	male	CCRCC_PRCC	synchronous	NA	NA	

333	29119638	57	male	multiple_PRCC	synchronous	NA	NA	
334	29119638	68	male	CCRCC_PRCC	synchronous	NA	NA	
335	29119638	54	male	multiple_PRCC	synchronous	NA	NA	unselected
336	29119638	53	male	PRCC_other	synchronous	NA	NA	
337	29119638	39	male	CCRCC_PRCC	synchronous	NA	NA	
338	28130179	NOS	NOS	CCRCC_other	synchronous	family history	NA	
339	28130179	NOS	NOS	CCRCC_other	synchronous	sporadic	NA	
340	28130179	NOS	NOS	PRCC_other	synchronous	sporadic	NA	
341	15964347	NOS	NOS	NOS	synchronous	NA	NA	
342	5839564	54	male	multiple_CCRCC	metachronou	NA	NA	
343	10433944	42	female	multiple_PRCC	synchronous	family history	MET	
344	10433944	37	female	multiple_PRCC	synchronous	family history	MET	
345	10433944	43	male	multiple_PRCC	synchronous	family history	MET	
346	10433944	60	female	multiple_PRCC	synchronous	family history	MET	
347	10433944	45	male	multiple_PRCC	synchronous	family history	MET	
348	10433944	55	male	multiple_PRCC	synchronous	family history	MET	
349	10433944	72	male	multiple_PRCC	synchronous	family history	MET	with germline MET variant only; papillary RCC only
350	10433944	50	female	multiple_PRCC	synchronous	family history	MET	
351	10433944	42	male	multiple_PRCC	synchronous	family history	MET	
352	10433944	48	male	multiple_PRCC	synchronous	family history	MET	
353	10433944	47	female	multiple_PRCC	synchronous	family history	MET	
354	10433944	33	female	multiple_PRCC	synchronous	family history	MET	
355	10433944	45	male	multiple_PRCC	synchronous	family history	MET	
356	10433944	69	female	multiple_PRCC	synchronous	family history	MET	
357	10433944	41	female	multiple_PRCC	synchronous	family history	MET	
358	10433944	NOS	NOS	multiple_PRCC	synchronous	sporadic	MET	
359	29062716	72	male	multiple_CCRCC	synchronous	NA	NA	
360	3288889	34	male	NOS	metachronou	NA	NA	
361	3288889	45	male	NOS	metachronou	NA	NA	
362	3288889	44	male	NOS	metachronou	NA	NA	
363	10083767	NOS	NOS	NOS	NA	NA	NA	
364	9796982	44	male	NOS	synchronous	NA	NA	
365	9796982	58	female	NOS	synchronous	NA	NA	
366	9796982	57	male	NOS	synchronous	NA	NA	
367	9796982	68	male	NOS	synchronous	NA	NA	
368	9796982	82	female	NOS	synchronous	NA	NA	unselected
369	9796982	61	female	NOS	synchronous	NA	NA	
370	9796982	47	male	NOS	synchronous	NA	NA	
371	9796982	63	male	NOS	synchronous	NA	NA	
372	9796982	59	female	NOS	synchronous	NA	NA	
373	9796982	71	male	NOS	synchronous	NA	NA	
374	9884257	NOS	NOS	multiple_PRCC	synchronous	NA	NA	

375	9884257	NOS	NOS	multiple_CCRCC	synchronous	NA	NA	
376	32133249	69	male	CCRCC_ChRCC	synchronous	NA	NA	
377	20279571	27	female	NOS	synchronous	NA	NA	
378	103400	45	male	NOS	synchronous	NA	NA	
379	103400	70	female	NOS	synchronous	NA	NA	
380	103400	61	female	NOS	synchronous	NA	NA	synchronous
381	103400	46	male	NOS	synchronous	NA	NA	only; bilateral
382	103400	82	male	NOS	synchronous	NA	NA	only
383	103400	51	male	NOS	synchronous	NA	NA	
384	103400	40	male	NOS	synchronous	NA	NA	
385	14069435	48	female	multiple_PRCC	synchronous	NA	NA	
386	5047402	38	female	NOS	metachronou	NA	NA	
387	3303609	57	male	multiple_CCRCC	synchronous	NA	NA	
388	3418791	54	NOS	multiple_CCRCC	synchronous	NA	NA	
389	3418791	54	NOS	multiple_CCRCC	synchronous	NA	NA	
390	3418791	76	NOS	multiple_CCRCC	synchronous	NA	NA	
391	3418791	61	NOS	multiple_CCRCC	synchronous	NA	NA	
392	660755	NOS	NOS	NOS	synchronous	NA	NA	
393	2925273	53	male	multiple_CCRCC	synchronous	family history	NA	
394	4033229	73	male	NOS	synchronous	NA	NA	
395	4033229	71	female	NOS	synchronous	NA	NA	
396	4033229	58	male	NOS	synchronous	NA	NA	
397	4033229	75	male	NOS	synchronous	NA	NA	
398	4033229	56	male	NOS	synchronous	NA	NA	
399	4033229	71	female	NOS	synchronous	NA	NA	
400	4033229	68	female	NOS	synchronous	NA	NA	bilateral only
401	4033229	80	male	NOS	synchronous	NA	NA	
402	4033229	66	male	NOS	synchronous	NA	NA	
403	4033229	56	male	NOS	synchronous	NA	NA	
404	4033229	63	male	NOS	metachronou	NA	NA	
405	4033229	46	male	NOS	metachronou	NA	NA	
406	4033229	55	male	NOS	metachronou	NA	NA	
407	27396730	65	male	multiple_CCRCC	metachronou	NA	NA	
408	33419828	NOS	NOS	NOS	synchronous	NA	NA	
409	14560563	NOS	NOS	multiple_CCRCC	NA	NA	NA	
410	14560563	NOS	NOS	NOS	NA	NA	NA	
411	14560563	NOS	NOS	hybrid	NA	NA	NA	
412	14560563	NOS	NOS	hybrid	NA	NA	NA	
413	480503	41	male	NOS	synchronous	NA	NA	
414	480503	37	female	NOS	synchronous	NA	NA	
415	480503	56	male	NOS	synchronous	NA	NA	
416	480503	35	female	NOS	metachronou	NA	NA	

417	11300946	53	male	PRCC_other	synchronous	NA	NA	
418	7460991	54	female	multiple_CCRCC	synchronous	NA	NA	
419	7460991	64	male	multiple_CCRCC	synchronous	NA	NA	
420	29066638	30	female	multiple_ChRCC	synchronous	NA	BHD	
421	628987	45	male	NOS	metachronou	NA	NA	
422	628987	48	female	NOS	metachronou	NA	NA	
423	628987	60	male	NOS	synchronous	NA	NA	
424	33165095	42	male	PRCC_other	synchronous	NA	NA	
425	21031096	43	female	CCRCC_ChRCC	synchronous	NA	TSC	
426	19403547	43	male	CCRCC_ChRCC	synchronous	family history	TSC	
427	33113122	39	NOS	hybrid	synchronous	sporadic	TSC	
428	27061669	70	male	CCRCC_other	metachronou	NA	NA	
429	33106702	47	female	NOS	metachronou	family history	VHL	
430	24696288	71	male	NOS	metachronou	sporadic	no	
431	24696288	67	male	NOS	metachronou	sporadic	no	
432	24696288	70	male	NOS	metachronou	sporadic	no	no hereditary
433	24696288	59	female	NOS	metachronou	sporadic	no	RCC syndrome;
434	24696288	77	male	NOS	metachronou	sporadic	no	metachronous
435	24696288	67	female	NOS	metachronou	sporadic	no	only; bilateral
436	24696288	73	male	NOS	metachronou	sporadic	no	only
437	24696288	84	male	NOS	metachronou	sporadic	no	
438	24696288	58	male	NOS	metachronou	sporadic	no	
439	4069171	78	female	NOS	synchronous	NA	NA	
440	16337860	43	male	hybrid	synchronous	NA	NA	
441	29267457	13	female	multiple_PRCC	metachronou	family history	HLRCC	
442	22218055	47	female	multiple_CCRCC	metachronou	NA	NA	
443	3404971	53	male	NOS	synchronous	NA	NA	
444	10196620	NOS	NOS	NOS	synchronous	NA	NA	
445	1394233	50	female	NOS	synchronous	NA	NA	
446	1394233	65	male	multiple_CCRCC	synchronous	NA	NA	
447	1394233	47	male	multiple_CCRCC	synchronous	NA	NA	
448	1394233	29	female	multiple_CCRCC	synchronous	NA	NA	
449	4061474	68	male	NOS			NA	
450	19616232	NOS	NOS	multiple_CCRCC	synchronous	NA	NA	
451	19616232	NOS	NOS	CCRCC_ChRCC	synchronous	NA	NA	
452	741549	28	male	CCRCC_PRCC	synchronous	NA	NA	
453	10478670	67	male	hybrid	synchronous	NA	NA	
454	10478670	79	female	multiple_oncocytoma	synchronous	NA	NA	
455	10478670	73	female	multiple_oncocytoma	metachronou	NA	NA	bilateral only;
456	10478670	62	male	multiple_oncocytoma	synchronous	NA	NA	oncocytoma only
457	10478670	34	female	multiple_oncocytoma	synchronous	NA	NA	
458	10478670	48	female	multiple_oncocytoma	metachronou	NA	NA	

459	26347846	47	male	other	synchronous	family history	NA	
460	3429189	18	female	other	synchronous	NA	TSC	
461	29193813	72	male	multiple_ChRCC			NA	
462	15577450	85	male	NOS	synchronous	NA	NA	
463	15577450	78	male	NOS	synchronous	NA	NA	
464	10417759	56	NOS	multiple_PRCC	NA	sporadic	NA	
465	10417759	28	NOS	multiple_PRCC	NA	family history	MET	
466	10417759	44	NOS	multiple_PRCC	NA	sporadic	NA	
467	10417759	71	NOS	multiple_PRCC	NA	sporadic	NA	
468	8558631	56	male	NOS	metachronou	NA	NA	
469	33654607	32	male	NOS	synchronous	family history	VHL	
470	33654607	34	male	NOS	synchronous	family history	VHL	
471	12560445	9	female	NOS	synchronous	NA	NA	
472	18308104	NOS	NOS	multiple_CCRCC	metachronou	NA	NA	
473	6827665	40	male	CCRCC_other	synchronous	NA	NA	
474	6827665	38	male	NOS	synchronous	NA	NA	
475	6827665	67	male	multiple_oncocytoma	synchronous	NA	NA	
476	6827665	63	male	NOS	synchronous	NA	NA	
477	12492948	NOS	NOS	CCRCC_other	synchronous	NA	NA	
478	18246052	45	female	hybrid	synchronous	NA	NA	
479	20924276	53	female	other	synchronous	NA	NA	
480	20924276	56	male	other	synchronous	NA	NA	
481	20924276	73	male	other	synchronous	NA	NA	
482	20924276	65	female	other	synchronous	NA	NA	
483	20924276	29	male	other	synchronous	NA	NA	clear cell
484	20924276	56	male	other	synchronous	NA	NA	tubulopapillary
485	20924276	53	male	other	synchronous	NA	NA	RCC only
486	20924276	57	male	other	synchronous	NA	NA	
487	20924276	61	female	other	synchronous	NA	NA	
488	20924276	78	male	other	synchronous	NA	NA	
489	20924276	49	female	other	synchronous	NA	NA	
490	17539022	47	female	multiple_CCRCC	synchronous	family history	chromosomal translocation	
491	17539022	44	male	multiple_CCRCC	synchronous	family history	chromosomal translocation	
492	32162575	NOS	NOS	multiple_oncocytoma	synchronous	NA	NA	
493	25034258	72	male	NOS	synchronous	sporadic	NA	
494	19921990	55	female	NOS	synchronous	NA	NA	
495	14501705	52	female	multiple_PRCC	synchronous	NA	NA	
496	17270241	23	female	CCRCC_PRCC	synchronous	family history	HLRCC	
497	30167812	NOS	NOS	multiple_CCRCC	synchronous	NA	NA	
498	28499369	55	male	multiple_ChRCC	synchronous	family history	BHD	
499	30066860	61	male	multiple_CCRCC	synchronous	NA	VHL	
500	30066860	39	female	multiple_CCRCC	synchronous	NA	VHL	

501	28697675	61	male	CCRCC_PRCC	synchronous	sporadic	NA	
502	29682611	47	female	multiple_CCRCC	synchronous	NA	NA	
503	945722	31	male	NOS	NA	family history	VHL	VHL only; familial only
504	945722	45	female	NOS	NA	family history	VHL	
505	945722	45	male	NOS	NA	family history	VHL	
506	945722	32	male	NOS	NA	family history	VHL	
507	945722	42	female	NOS	NA	family history	VHL	
508	23384228	65	female	NOS	synchronous	family history	VHL	
509	9313655	46	male	NOS	synchronous	NA	VHL	
510	9313655	31	female	NOS	synchronous	NA	VHL	
511	9313655	50	male	NOS	synchronous	NA	VHL	
512	12459621	39	male	hybrid	NA	family history	BHD	BHD only; familial only
513	12459621	39	male	hybrid	NA	family history	BHD	
514	12459621	44	male	multiple_ChRCC	NA	family history	BHD	
515	12459621	31	male	hybrid	NA	family history	BHD	
516	12459621	55	male	hybrid	NA	family history	BHD	
517	12459621	52	male	hybrid	NA	family history	BHD	
518	12459621	48	female	multiple_ChRCC	NA	family history	BHD	
519	12459621	64	male	CCRCC_other	NA	family history	BHD	
520	12459621	54	male	multiple_CCRCC	NA	family history	BHD	
521	12459621	39	male	multiple_ChRCC	NA	family history	BHD	
522	12459621	65	male	multiple_ChRCC	NA	family history	BHD	
523	12459621	67	male	CCRCC_ChRCC	NA	family history	BHD	
524	12459621	45	male	multiple_CCRCC	NA	family history	BHD	
525	12459621	61	male	CCRCC_ChRCC	NA	family history	BHD	
526	12459621	46	male	multiple_ChRCC	NA	family history	BHD	
527	12459621	69	female	hybrid	NA	family history	BHD	
528	12459621	48	male	multiple_ChRCC	NA	family history	BHD	
529	12459621	37	male	CCRCC_ChRCC	NA	family history	BHD	
530	12459621	60	female	hybrid	NA	family history	BHD	
531	12459621	57	male	multiple_ChRCC	NA	family history	BHD	
532	12459621	35	female	multiple_ChRCC	NA	family history	BHD	
533	12459621	61	male	multiple_ChRCC	NA	family history	BHD	
534	24772173	44	male	multiple_ChRCC	synchronous	family history	BHD	
535	15599549	68	female	NOS	synchronous	NA	NA	
536	21883816	68	male	multiple_PRCC	synchronous	sporadic	NA	non-familial only
537	21883816	74	female	multiple_CCRCC	synchronous	sporadic	NA	
538	21883816	74	male	NOS	synchronous	sporadic	NA	
539	21883816	83	male	multiple_PRCC	synchronous	sporadic	NA	
540	21883816	61	male	multiple_PRCC	synchronous	sporadic	NA	
541	21883816	66	male	multiple_PRCC	synchronous	sporadic	NA	

542	4583817	45	male	NOS	synchronous	family history	NA	
543	17349574	73	female	multiple_oncocytoma	synchronous	sporadic	NA	
544	8952541	30	male	NOS	synchronous	family history	VHL	
545	8952541	39	male	NOS	synchronous	family history	VHL	
546	28188682	NOS	NOS	NOS	NA	NA	NA	
547	11776380	45	male	multiple_CCRCC	synchronous	family history	NA	
548	11776380	38	male	multiple_CCRCC	synchronous	family history	NA	
549	11776380	51	male	NOS	NA	family history	NA	
550	22458491	NOS	NOS	NOS	NA	NA	NA	
551	26342594	46	female	multiple_ChRCC	synchronous	family history	BHD	
552	25025441	34	male	NOS	metachronou	sporadic	SDH	
553	25025441	14	male	NOS	metachronou	family history	SDH	
554	25025441	27	male	NOS	synchronous	sporadic	SDH	
555	34538873	63	female	multiple_ChRCC	synchronous	NA	NA	
556	6823722	43	female	NOS	synchronous	family history	VHL	
557	6823722	37	male	NOS	synchronous	family history	VHL	
558	3761431	28	male	multiple_CCRCC	synchronous	NA	VHL	
559	2815455	60	male	NOS	synchronous	family history	VHL	
560	34414496	36	NOS	NOS	NA	NA	VHL	VHL only
561	34414496	45	NOS	NOS	NA	NA	VHL	
562	34414496	45	NOS	NOS	NA	NA	VHL	
563	34414496	62	NOS	NOS	NA	NA	VHL	
564	34414496	29	NOS	NOS	NA	NA	VHL	
565	34414496	61	NOS	NOS	NA	NA	VHL	
566	34414496	51	NOS	NOS	NA	NA	VHL	
567	34414496	53	NOS	NOS	NA	NA	VHL	
568	18074239	68	female	NOS	NA	family history	VHL	
569	25159823	32	male	multiple_CCRCC	synchronous	NA	VHL	
570	3812515	31	male	NOS	metachronou	family history	VHL	
571	3812515	45	male	NOS	synchronous	family history	VHL	
572	3812515	43	female	NOS	synchronous	family history	VHL	
573	3812515	30	male	NOS	metachronou	sporadic	VHL	
574	33425683	75	female	multiple_CCRCC	synchronous	NA	VHL	
575	19715686	37	female	multiple_CCRCC	synchronous	family history	VHL	
576	19715686	78	male	multiple_CCRCC	synchronous	family history	VHL	
577	30291511	14	male	NOS	synchronous	NA	VHL	
578	26622630	33	male	multiple_CCRCC	synchronous	sporadic	VHL	
579	31930078	NOS	female	multiple_CCRCC	synchronous	family history	VHL	
580	15931650	45	female	NOS	NA	NA	VHL	
581	7974953	37	male	multiple_CCRCC	synchronous	NA	VHL	VHL only; bilateral only
582	7974953	31	male	NOS	synchronous	sporadic	VHL	
583	7974953	33	male	multiple_CCRCC	synchronous	family history	VHL	

584	7974953	47	male	NOS	synchronous	family history	VHL	bilateral only
585	7974953	20	male	multiple_CCRCC	synchronous	NA	VHL	
586	17268895	65	male	multiple_CCRCC	synchronous	sporadic	VHL	
587	7314362	28	male	NOS	synchronous	NA	VHL	
588	7441848	39	male	NOS	synchronous	family history	VHL	
589	7441848	28	male	NOS	synchronous	family history	VHL	
590	7441848	24	female	NOS	NA	sporadic	VHL	
591	15198146	48	male	other	metachronou	NA	VHL	
592	11688394	45	male	NOS	synchronous	family history	VHL	
593	1593671	28	NOS	multiple_CCRCC	synchronous	NA	VHL	VHL only;bilateral only; 5 had family history
594	1593671	30	NOS	multiple_CCRCC	synchronous	NA	VHL	
595	1593671	26	NOS	multiple_CCRCC	synchronous	NA	VHL	
596	1593671	67	NOS	multiple_CCRCC	synchronous	NA	VHL	
597	1593671	45	NOS	multiple_CCRCC	synchronous	NA	VHL	
598	1593671	31	NOS	multiple_CCRCC	synchronous	NA	VHL	
599	1593671	39	NOS	multiple_CCRCC	synchronous	NA	VHL	
600	1593671	26	NOS	multiple_CCRCC	synchronous	NA	VHL	
601	1593671	29	NOS	multiple_CCRCC	synchronous	NA	VHL	
602	327975	30	female	NOS	metachronou	family history	VHL	
603	15772467	NOS	female	multiple_CCRCC	synchronous	family history	VHL	
604	8493465	27	female	multiple_CCRCC	synchronous	family history	VHL	
605	8493465	23	male	NOS	synchronous	family history	VHL	
606	8493465	23	male	NOS	metachronou	family history	VHL	
607	8493465	30	female	multiple_CCRCC	synchronous	NA	VHL	
608	30430496	36	female	NOS	synchronous	sporadic	VHL	
609	850326	34	male	multiple_CCRCC	synchronous	family history	VHL	
610	11844068	45	female	multiple_CCRCC	synchronous	sporadic	VHL	
611	26511773	44	male	multiple_CCRCC	synchronous	NA	VHL	
612	17657389	39	male	NOS	synchronous	family history	VHL	
613	2397466	38	female	NOS	NA	family history	VHL	
614	2397466	39	female	NOS	synchronous	family history	VHL	
615	26812297	60	female	multiple_CCRCC	synchronous	sporadic	VHL	
616	28650600	47	female	CCRCC_other	synchronous	NA	VHL	
617	675929	36	male	NOS	synchronous	NA	VHL	
618	20442526	56	NOS	multiple_CCRCC	synchronous	sporadic	VHL	
619	11284029	54	female	NOS	NA	NA	VHL	VHL only
620	11284029	42	male	NOS	NA	NA	VHL	
621	11284029	62	male	NOS	NA	NA	VHL	
622	11284029	27	female	NOS	NA	NA	VHL	
623	11284029	38	female	NOS	NA	NA	VHL	
624	11284029	39	male	NOS	NA	NA	VHL	
625	26425233	38	female	multiple_CCRCC	synchronous	family history	VHL	

626	27123102	17	male	multiple_CCRCC	synchronous	sporadic	VHL	
627	23298237	34	male	NOS	synchronous	family history	VHL	
628	18485044	NOS	NOS	NOS	synchronous	NA	VHL	
629	18485044	NOS	NOS	NOS	synchronous	NA	VHL	
630	18485044	NOS	NOS	NOS	synchronous	NA	VHL	
631	18485044	NOS	NOS	NOS	metachronou	NA	VHL	
632	32179488	44	male	multiple_CCRCC	synchronous	family history	VHL	
633	32179488	48	male	multiple_CCRCC	synchronous	family history	VHL	
634	32179488	63	female	multiple_CCRCC	metachronou	sporadic	VHL	
635	32742360	24	male	NOS	NA	family history	VHL	
636	32742360	66	male	NOS	NA	family history	VHL	
637	32742360	34	male	NOS	NA	family history	VHL	
638	8245249	61	male	multiple_ChRCC	synchronous	family history	BHD	
639	30326848	29	female	multiple_ChRCC	synchronous	sporadic	BHD	
640	29719489	67	female	multiple_ChRCC	synchronous	NA	BHD	
641	25757955	21	male	multiple_CCRCC	synchronous	NA	TSC	
642	9827727	7	male	NOS	synchronous	sporadic	TSC	
643	9179674	42	male	multiple_ChRCC	synchronous	sporadic	TSC	
644	6502814	23	female	multiple_CCRCC	synchronous	family history	TSC	
645	25432535	24	female	multiple_PRCC	synchronous	NA	TSC	
646	25432535	NOS	male	multiple_PRCC	synchronous	sporadic	TSC	
647	18728283	30	female	NOS	NA	sporadic	SDH	
648	18728283	46	male	multiple_ChRCC	NA	sporadic	SDH	
649	22351710	68	female	CCRCC_PRCC	metachronou	family history	SDH	
650	28165631	35	male	other	synchronous	NA	HLRCC	
651	28165631	40	male	other	synchronous	family history	HLRCC	
652	9140397	56	NOS	multiple_PRCC	synchronous	sporadic	MET	
653	9140397	NOS	NOS	multiple_PRCC	NA	family history	MET	
654	34801057	51	male	CCRCC_PRCC	synchronous	sporadic	MET	
655	34949766	22	female	NOS	synchronous	NA	SDH	
656	34949766	19	male	NOS	metachronou	NA	NA	
657	34949766	50	male	NOS	synchronous	NA	SDH	SDH-deficient RCC only
658	34949766	72	female	NOS	synchronous	NA	NA	
659	34949766	37	male	NOS	metachronou	NA	NA	
660	35085167	26	female	NOS	synchronous	NA	NA	
661	35272357	45	NA	NOS	NA	NA	NA	
662	35272357	62	NA	NOS	NA	NA	NA	
663	35272357	74	NA	NOS	NA	NA	NA	
664	35272357	29	NA	NOS	NA	NA	NA	
665	35350482	66	male	multiple_oncocytoma	synchronous	family history	BHD	
666	35469800	51	male	multiple_CCRCC	synchronous	family history	NA	
667	35469800	36	male	multiple_CCRCC	synchronous	family history	NA	

668	35469800	54	female	multiple_CCRCC	synchronous	sporadic	NA	
669	35469800	62	male	multiple_CCRCC	synchronous	family history	NA	
670	35469800	39	male	multiple_CCRCC	synchronous	sporadic	NA	
671	35469800	59	male	multiple_CCRCC	synchronous	sporadic	NA	
672	35469800	76	female	multiple_CCRCC	metachronou	family history	NA	clear cell RCC
673	35469800	37	female	multiple_CCRCC	synchronous	sporadic	NA	only
674	35469800	59	male	multiple_CCRCC	metachronou	sporadic	NA	
675	35469800	42	male	multiple_CCRCC	metachronou	sporadic	NA	
676	35469800	36	female	multiple_CCRCC	synchronous	sporadic	NA	
677	35469800	46	male	multiple_CCRCC	synchronous	sporadic	NA	
678	35469800	41	male	multiple_CCRCC	synchronous	family history	NA	
679	35469800	36	male	multiple_CCRCC	synchronous	sporadic	NA	
680	35782879	84	male	multiple_CCRCC	synchronous	NA	NA	
681	35812682	72	male	CCRCC_PRCC	synchronous	sporadic	NA	
682	35924708	65	female	multiple_CCRCC	synchronous	NA	NA	
683	35924708	56	male	multiple_CCRCC	synchronous	NA	NA	
684	36002896	60	male	multiple_PRCC	NA	NA	NA	
685	36118792	41	female	multiple_PRCC	synchronous	family history	HLRCC	
686	36196127	53	male	CCRCC_PRCC	synchronous	NA	NA	
687	36204593	61	male	multiple_CCRCC	metachronou	NA	NA	
688	36258004	51	female	hybrid	synchronous	sporadic	BHD	
689	36261952	58	male	NOS	synchronous	NA	NA	
690	36291753	76	male	multiple_ChRCC	metachronou	NA	BHD	
691	36291753	84	male	other	synchronous	NA	BHD	
692	36291753	52	female	CCRCC_ChRCC	synchronous	NA	BHD	BHD only
693	36291753	64	male	multiple_PRCC	metachronou	NA	BHD	
694	36291753	59	male	multiple_ChRCC	synchronous	NA	BHD	
695	36291753	68	male	multiple_CCRCC	metachronou	NA	BHD	
696	36299391	35	female	NOS	synchronous	NA	NA	
697	36316043	67	female	multiple_CCRCC	synchronous	family history	chromosomal translocation	
698	36330476	25	female	multiple_ChRCC	synchronous	sporadic	HLRCC	
699	36381238	13	male	other	synchronous	NA	NA	
700	36611089	27	male	multiple_CCRCC	synchronous	family history	NA	
701	36685138	63	male	multiple_CCRCC	synchronous	NA	NA	
702	36687224	59	male	CCRCC_PRCC	synchronous	NA	NA	
703	36716731	67	male	multiple_CCRCC	synchronous	NA	NA	
704	37076922	60	male	NOS	synchronous	sporadic	HLRCC	
705	37006053	32	male	other	synchronous	NA	NA	
706	9461085	68	female	multiple_CCRCC	synchronous	family history	chromosomal translocation	
707	9461085	54	male	multiple_CCRCC	metachronou	family history	chromosomal translocation	
708	38440695	29	male	mucinous tubular and sp	metachronou	family history	HLRCC	
709	38322096	41	female	multiple_CCRCC	synchronous	NA	VHL	

710	38322096	55	male	hybrid	synchronous	NA	NA
711	38322096	60	male	CCRCC_NOS	metachronou	NA	NA
712	38241777	55	female	multiple_oncocytoma	synchronous	NA	no
713	38241777	75	female	multiple_oncocytoma	synchronous	NA	no
714	38196308	52	male	multiple_CCRCC	synchronous	NA	NA
715	38146729	29	NA	multiple_CCRCC	synchronous	family history	VHL
716	38146729	24	NA	multiple_CCRCC	synchronous	family history	VHL
717	38146729	32	NA	multiple_CCRCC	synchronous	family history	VHL
718	38146729	24	NA	multiple_CCRCC	synchronous	family history	VHL
719	38146729	54	NA	multiple_CCRCC	synchronous	family history	VHL
720	37994665	NA	NA	hybrid	NA	NA	BHD
721	37965647	47	male	other	synchronous	sporadic	no
722	37928302	44	male	CCRCC_PRCC	synchronous	NA	NA
723	37924432	60	male	NOS	metachronou	NA	NA
724	37844561	56	male	multiple_CCRCC	synchronous	NA	VHL
725	37844334	57	male	CCRCC_PRCC	synchronous	NA	NA
726	37823048	28	female	multiple_CCRCC	synchronous	sporadic	VHL
727	37811540	57	male	CCRCC_oncocytoma	synchronous	family history	NA
728	37715637	60	male	CCRCC_PRCC	synchronous	NA	HLRCC
729	37486723	36	male	multiple_oncocytoma	metachronou	NA	NA
730	37468236	NA	male	multiple_PRCC	synchronous	family history	HLRCC
731	37335313	66	male	PRCC_ChRCC	metachronou	NA	no
