# Supplementary material for: Characteristics, aetiology and implications for management of multiple primary renal tumours: a systematic review
Source: Eur J Hum Genet. 2024 May 27;32(8):887–94. doi: 10.1038/s41431-024-01628-5 (PMC11291654; doi:10.1038/s41431-024-01628-5)
Supplement: Supplementary file 5 — Supplementary Table 3 [file 41431_2024_1628_MOESM5_ESM.pdf]

**Supplementary Table 3. Examples of the renal cell carcinoma (RCC) susceptibility gene panels used for genetic testing**

| Invitae Renal/Urinary Tra | Hereditary Renal Cance | GeneDx Renal Cancer Pancel |
|---|---|---|
| CDKN1C (11p15.4) | CDKN1C (11p15.4) | BAP1 |
| DIS3L2 (2q37.1) | FLCN (17p11.2) | EPCAM |
| FLCN (17p11.2) | FH (1q43) | FH |
| FH (1q43) | GPC3 (Xq26.2) | FLCN |
| DICER1 (14q32.13) | MET (7q31.2) | MET |
| GPC3 (Xq26.2) | SDHAF2 (11q12.2) | MITF |
| MSH6 (2p16.3) | PTEN (10q23.31) | MLH1 |
| EPCAM (2p21) | SDHA (5p15.33) | MSH2 |
| MET (7q31.2) | SDHB (1p36.13) | MSH6 |
| MLH1 (3p22.2) | SDHC (1q23.3) | PMS2 |
| MSH2 (2p21-16.3) | SDHD (11q23.1) | PTEN |
| PMS2 (7p22.1) | VHL (3p25.3) | SDHB |
| PTEN (10q23.31) | WT1 (11p13) | SDHC |
| SDHB (1p36.13) | | SDHD |
| SDHC (1q23.3) | | TP53 |
| SMARCA4 (19p13.2) | | TSC1 |
| SMARCB1 (22q11.23) | | TSC2 |
| TP53 (17p13.1) | | VHL |
| TSC1 (9q34.13) | | |
| TSC2 (16p13.3) | | |
| VHL (3p25.3) | | |
| WT1 (11p13) | | |
| CDC73 (1q31.2) | | |
| BAP1 (3p21.1) | | |
